# A novel tetratricopeptide-repeat protein, TTP1, forms complexes with glutamyl-tRNA reductase and protochlorophyllide oxidoreductase during tetrapyrrole biosynthesis

**DOI:** 10.1093/jxb/erad491

**Published:** 2023-12-09

**Authors:** Josephine Herbst, Xiaoqing Pang, Lena Roling, Bernhard Grimm

**Affiliations:** Humboldt-Universität zu Berlin, Institute of Biology—Plant Physiology, Philippstr. 13, Building 12, 10099 Berlin, Germany; VIB-U Gent Center for Plant Systems Biology, Ghent University, Technologiepark-Zwijnaarde 71, 9052 Ghent, Belgium; Humboldt-Universität zu Berlin, Institute of Biology—Plant Physiology, Philippstr. 13, Building 12, 10099 Berlin, Germany; Humboldt-Universität zu Berlin, Institute of Biology—Plant Physiology, Philippstr. 13, Building 12, 10099 Berlin, Germany; Humboldt-Universität zu Berlin, Institute of Biology—Plant Physiology, Philippstr. 13, Building 12, 10099 Berlin, Germany; Bielefeld University, Germany

**Keywords:** 5-Aminolevulinic acid synthesis, chlorophyll, chloroplast biogenesis, chloroplast metabolism, post-translational control, protein–protein interaction, tetrapyrrole biosynthesis, tetratricopeptide-repeat proteins

## Abstract

The biosynthesis of the tetrapyrrole end-products chlorophyll and heme depends on a multifaceted control mechanism that acts primarily at the post-translational level upon the rate-limiting step of 5-aminolevulinic acid synthesis and upon light-dependent protochlorophyllide oxidoreductase (POR). These regulatory processes require auxiliary factors that modulate the activity, stability, complex formation, and subplastidal localization of the relevant proteins. Together, they ensure optimal metabolic flow during the day and at night. As an Arabidopsis homolog of the POR-interacting tetratricopeptide-repeat protein (Pitt) first reported in *Synechocystis*, we characterize tetrapyrrole biosynthesis-regulating tetratricopeptide-repeat protein1 (TTP1). TTP1 is a plastid-localized, membrane-bound factor that interacts with POR, the Mg protoporphyrin monomethylester cyclase CHL27, glutamyl-tRNA reductase (GluTR), GluTR-binding protein, and FLUORESCENCE IN BLUE LIGHT. Lack of TTP1 leads to accumulation of GluTR, enhanced 5-aminolevulinic acid synthesis and lower levels of POR. Knockout mutants show enhanced sensitivity to reactive oxygen species and a slower greening of etiolated seedlings. Based on our studies, the interaction of TTP1 with GluTR and POR does not directly inhibit their enzymatic activity and contribute to the control of 5-aminolevulinic acid synthesis. Instead, we propose that TTP1 sequesters a fraction of these proteins on the thylakoid membrane, and contributes to their stability.

## Teaser

A novel Arabidopsis tetratricopeptide-repeat protein, TTP1, anchors three enzymes of chlorophyll synthesis and the regulator GluTR-binding protein to the plastid membrane to support the inhibition of GluTR by the negative regulator FLUORESCENCE IN BLUE LIGHT.

## Introduction

All living organisms on Earth depend on the availability of tetrapyrroles—a class of macrocyclic components with essential functions in many biochemical and photophysical processes (Tanaka and Tanaka, 2007). Oxygenic photoautotrophs utilize a common multi-enzymatic metabolic pathway to synthesize a set of tetrapyrrolic end-products, and eukaryotic photoautotrophs use the resulting chlorophylls (Chl *a* and *b*), siroheme, heme, and bilins in a variety of contexts (Grimm, 2019).

In many bacteria and all oxygenic photoautotrophs, 5ʹ-aminolevulinic acid (ALA), the first metabolite that is specific to tetrapyrrole biosynthesis (TBS), is synthesized from activated glutamate. The activation of glutamate is performed by ligation to a transfer-RNA(Glu) (tRNA^glu^). In the first committed step in TBS (Tanaka *et al.*, 2011), glutamyl-tRNA reductase (GluTR) then converts the glutamate into glutamate-1-semialdehyde. In Arabidopsis, GluTR is encoded by three genes. *HEMA1* is predominantly expressed in green leaves, *HEMA2* has a low basal expression, and *HEMA3* seems to be a pseudogene (Matsumoto *et al.*, 2004). Glutamate-1-semialdehyde is subsequently transaminated to ALA by glutamate-1-semialdehyde aminotransferase (GSAAT) (Mayer and Beale, 1990).

**Fig. 1.**
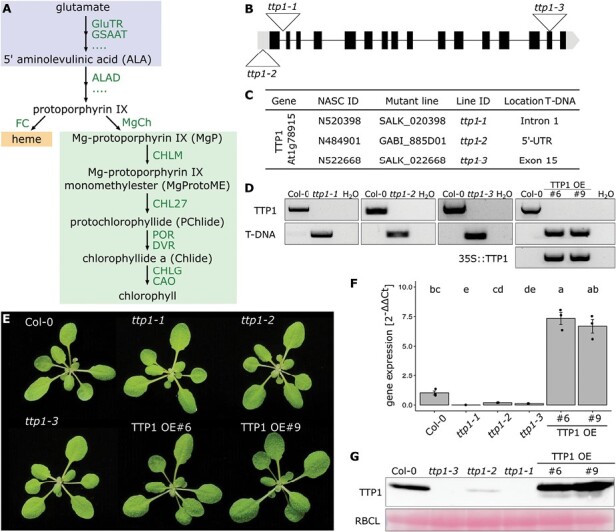
Identification and selection of *TTP1* knockout and knockdown mutants and overexpression lines. (A) Simplified depiction of the tetrapyrrole biosynthesis (TBS) pathway in higher plants, highlighting the enzymatic steps that are mentioned in the text and are relevant for the elucidation of the function of TTP1. ALAD, 5-aminolevulinc acid dehydratase; CAO, chlorophyll *a* oxygenase; CHL27, catalytic subunit of Mg protoporphyrin monomethylester cyclase; CHLG, chlorophyll synthase; CHLM, Mg protoporphyrin methyltransferase; DVR, divinyl reductase; FC, ferrochelatase; GluTR, glutamyl-tRNA reductase; GSAAT, glutamate-1-semialdehyde aminotransferase; MgCh, magnesium chelatase; MgP, Mg protoporphyrin IX; POR, protochlorophyllide oxidoreductase. (B) Three independent T-DNA insertion lines for the TTP1-encoding gene *At1g78915* were selected for further analyses. Each of the three T-DNA insertion sites is depicted by a white triangle. (C) Overview of the T-DNA insertion lines used in this study, which were obtained from the Nottingham Arabidopsis Stock Centre (NASC). (D) The homozygous insertion of each of the T-DNAs was verified by PCR. The T-DNA insertion line *ttp1-3* was successfully complemented by the full-length coding sequence of TTP1 (followed by a HA-Strep tag), which was expressed under the control of the CaMV 35S promoter. The homozygous insertion of the T-DNA into the TTP1-encoding gene *At1g78915*, as well as the integration of the p35S:TTP1-HA cassette into the genome of the *ttp1-3* background, was verified by PCR. The endogenous *TTP1* gene was amplified from wild-type seedlings (Col-0) and served as a positive control for the intactness of the endogenous *TTP1* gene, while H_2_O was used as negative control. (E) Phenotypes of representative plants bearing each of the three T-DNA insertion lines and two independent TTP1 overexpression lines. Seedlings were grown for 3 weeks under short-day conditions (SD, 10 h light–14 h darkness, 120 µmol photons m^−2^ s^−1^, 21 °C, 60% humidity). (F) Levels of *TTP1* expression in the selected mutant and overexpression lines in comparison with the wild type. *SAND* was selected as the reference gene. Statistical significance was assessed with the Kruskal–Wallis test (Bonferroni post-hoc test, *P*≤0.05, *n*=3). Different lowercase letters above the bars indicate statistically significant differences (*P*<0.05). (G) The TTP1 protein content in the selected transgenic lines was determined by using a TTP1 antibody. The large subunit of RuBisCO (RBCL) was used as a loading control.

Two molecules of ALA are condensed to form the monopyrrole porphobilinogen, before the linear tetrapyrrole intermediate hydroxymethylbilane is formed in a stepwise manner. Subsequently, this molecule is folded into a porphyrin macrocycle and processed to yield protoporphyrin IX. Protoporphyrin IX is either chelated with ferrous iron by ferrochelatase to form protoheme, or with the divalent magnesium cation by magnesium chelatase to form Mg protoporphyrin IX. At least five subsequent enzymatic steps are needed to convert Mg protoporphyrin IX into Chl *a*, some of which can be transformed into Chl *b* and recycled to Chl *a* and chlorophyllide (Chlide) *a* via the chlorophyll cycle (Tanaka *et al.*, 2011) (Fig. 1A).

ALA synthesis is considered to be the rate-limiting step of TBS. Demands for heme and chlorophyll vary during photoperiodic growth and under fluctuating environmental conditions. Furthermore, the dependence of protochlorophyllide (PChlide) oxidoreductase (POR) on light precludes ALA synthesis in the dark. This in turn necessitates rapid and tight spatiotemporal control of the supply of ALA for the tetrapyrrole end-products. Moreover, owing to their photoreactive nature, transient accumulation of TBS intermediates is deleterious, as these pigments can readily generate excessive amounts of reactive oxygen species (ROS).

Higher plants have evolved multiple post-translational control mechanisms at specific checkpoints in the TBS pathway, such as ALA synthesis, Mg chelation of protoporphyrin IX, and PChlide reduction (Wang and Grimm, 2021). Several factors control the stability, activity, and subcompartmental localization of GluTR and its interaction with GSAAT. The GluTR-binding protein (GBP) not only protects GluTR from proteolysis by the caseinolytic protease (Clp) (Apitz *et al.*, 2016), it also stabilizes the association between GluTR and GSAAT in a multimeric protein complex (Sinha *et al.*, 2022). Conversely, the binding of GBP to GluTR is attenuated by a heme-dependent process (Richter *et al*., 2019). When heme binds to GBP, the GBP–GluTR interaction is destabilized, which exposes GluTR to proteolytic degradation (Richter *et al*., 2019). This mechanism therefore confirms the long-standing hypothesis of a heme-dependent, post-translational suppression of ALA synthesis.

In addition, chloroplast signal recognition particle 43 (cpSRP43) acts as a chaperone to prevent the aggregation and inactivation of GluTR by binding to the N-terminus of the enzyme, which contains two aggregation-prone regions (Wang *et al.*, 2018). Finally, ALA synthesis is repressed in the dark, and under unfavorable environmental conditions, by the interaction of the dominant GluTR isoform (GluTR1), with the membrane-localized protein FLUORESCENCE IN BLUE LIGHT (FLU) (Meskauskiene *et al.*, 2001; Goslings *et al.*, 2004; Richter *et al.*, 2010; Kauss *et al.*, 2012; Zhang *et al.*, 2015; Hou *et al.*, 2019). Accumulating PChlide is sensed by inactive POR, which causes FLU to form a complex with GluTR1 at the thylakoid membrane (Kauss *et al.*, 2012; Schmied *et al.*, 2018). The mature FLU protein consists of an N-terminal transmembrane domain, a coiled-coil motif and a C-terminal tetratricopeptide-repeat (TPR) domain with three TPR motifs that mediate selective interactions with other proteins (Zhang *et al.*, 2015; Fang *et al.*, 2016). Thus, in addition to GluTR1, FLU physically interacts with both POR and CHL27, the catalytic subunit of the Mg protoporphyrin monomethylester oxidative cyclase (Kauss *et al.*, 2012). Indeed, the ability to suppress ALA synthesis was a prerequisite for the emergence of the light-dependent POR during the evolution of angiosperms (Wang *et al.*, 2022). The FLU-mediated inactivation of GluTR minimizes the supply of precursors for chlorophyll synthesis and prevents excess accumulation of PChlide (Kauss *et al.*, 2012). It has been proposed that the inactivation of GluTR by FLU is triggered by the binding of PChlide to POR.

Arabidopsis possesses three differentially expressed *POR* genes, namely *PORA*, *PORB*, and *PORC* (Gabruk *et al.*, 2015). *PORA* encodes the dominant isoform during skotomorphogenesis, *PORB* is continuously expressed in light and darkness, while *PORC* is light-induced in photosynthetically active tissues (Armstrong *et al.*, 1995; Frick *et al.*, 2003). In addition to binding to FLU, the POR enzymes in Arabidopsis interact with the chaperone-like protein of POR1 (CPP1) (Lee *et al.*, 2013), while POR associates with POR-interacting TPR protein (Pitt) in *Synechocystis* (Schottkowski *et al.*, 2009; Rengstl *et al.*, 2011). Pitt stabilizes POR on the thylakoid membranes, especially in the biogenesis centers of *Synechocystis*, where newly synthesized chlorophyll molecules are inserted into nascent photosynthetic subunits during their integration into the thylakoid membrane (Rast *et al.*, 2015, 2019).

Interestingly, both FLU and Pitt belong to a large class of TPR-containing proteins that participate in the fine-tuning of various biological processes (Bohne *et al.*, 2016). In chloroplasts, TPR proteins are involved in the regulation of gene expression, import processes, and the assembly and maintenance of the photosynthetic apparatus (Bohne *et al.*, 2016). The TPR motif that characterizes all members of this protein family consists of a stretch of 34 amino acids, which can include both small and large hydrophobic residues at only loosely fixed positions, and folds into two or three antiparallel helices (Andrade *et al.*, 2001; Zeytuni and Zarivach, 2012). Among the classes of proteins with repetitive peptide motifs, TPR proteins are thought to be the most ancient, because they are found in almost all investigated archaeal and bacterial species (Ponting *et al.*, 1999; D’Andrea and Regan, 2003). TPR proteins generally bind to other polypeptides that are associated with larger protein complexes (Phillips *et al.*, 2012).

In this study, we searched for an Arabidopsis homolog of Pitt that performs a comparable function in higher plants. Owing to the variability in the sequences of TPR motifs, and the size of the TPR protein family in plants, a definitive Pitt-like homolog was identified among several members of plastid-localized TPR proteins in Arabidopsis. The selected Pitt-like candidate is encoded in the gene locus *At1g78915* and is designated as TETRAPYRROLE BIOSYNTHESIS-REGULATING TETRATRICOPEPTIDE-REPEAT-PROTEIN1 (TTP1) here. TTP1 and Pitt share a membrane-spanning domain and a domain with five canonical TPR motifs. We report the functional analysis of TTP1, which turns out to play a role in the assignment of GluTR to the thylakoid membrane and the FLU-dependent suppression of ALA synthesis.

## Materials and methods

### Plant material and growth regime

Arabidopsis plants were grown on soil under short days (10 h light/14 h darkness; 120 µmol photons m^−2^ s^−1^; 22 °C). Three T-DNA insertion lines, *ttp1-1* (SALK_020398), *ttp1-2* (GABI_885D01), and *ttp1-3* (SALK_022668), were obtained from the Nottingham Arabidopsis Stock Centre. The homozygous insertion of the T-DNA in the TTP1-encoding gene *At1g78915* was verified by genotyping (primers are listed in Supplementary Table S1). The Arabidopsis lines were subjected to various light regimes—continuous light (120 µmol photons m^−2^ s^−1^, 21 °C), long day (16 h light/8 h darkness; 120 µmol photons m^−2^ s^−1^; 21 °C), or fluctuating light (15 min 3 µmol photons m^−2^ s^−1^, 15 min 300 µmol photons m^−2^ s^−1^, 21 °C). For etiolation, seeds were subjected to 1 h of white light and grown for 6 d in the dark.

### RNA isolation and qRT-PCR

Total RNA was isolated from leaf material according to Onate-Sanchez and Vicente-Carbajosa (2008), omitting the sodium acetate precipitation. Then 1 µg of total RNA was treated with 1 U DNase I (Thermo Fisher Scientific) and cDNA synthesis was performed with RevertAid (Thermo Fisher Scientific) according to manufacturer’s instructions. To quantify gene-expression levels by qRT-PCR, 2× SYBR Green qPCR Master Mix (Biotool) was used according to manufacturer's instructions. All expression levels were normalized with the expression of *SAND* (*At2g28390*), in a few cases additionally with 5ʹ*GAPDH* (*At1g13440*). Primers are given in Supplementary Table S1.

### Generation of antibodies against TTP1

The coding sequence of *TTP1*, without transit peptide, was amplified with specific primers (Supplementary Table S1) from wild-type cDNA. Addition of *Bam*HI and *Sal*I restriction sites enabled ligation of the *TTP1* gene into the pET-28a(+) vector with a C-terminal 6×-histidine tag. This vector was transformed into Rosetta 2 (DE3) *Escherichia coli* cells, and recombinant expression of the TTP1 protein was induced with 0.5 mM isopropyl-β-d-thiogalactopyranoside. After incubation of the induced *E. coli* culture for 3 h at 37 °C, the recombinant protein was harvested and purified under native conditions on Ni-nitrilotriacetic acid agarose according to manufacturer's instructions (Qiagen, Germany). In total 1 µg of purified protein was used for immunization of two rabbits. The immunization was performed by BioGenes (Germany). The antisera obtained were used directly for immunoblotting.

### Pigment isolation and HPLC analysis

Pigments and tetrapyrrole intermediates were isolated with 0.5 ml basic acetone (acetone: 0.2 M NH_4_OH; 9:1) from lyophilized leaf material (25–30 mg). Samples were incubated for 20 min at –20 °C and centrifuged for 30 min (4 °C, 16 100×g). The clear supernatant was analysed by HPLC as previously described (Richter *et al*., 2019).

### 5-Aminolevulinic acid synthesis measurement

Leaf discs were submerged in 50 mM Tris–HCl buffer (pH 7.2) containing 40 mM levulinic acid and incubated for 4 h in the light (120 µmol photons m^–2^ s^–1^; 22 °C). The ALA content was determined as described previously (Mauzerall and Granick, 1956) and normalized to fresh or dry weight.

### Protein extraction and western blot analysis

Levels of tetrapyrrole-related proteins were determined in either total leaf extracts or subfractionated leaf material. For isolation of total leaf extracts, the lyophilized material was mixed with 10 µl mg^−1^ 2× Laemmli sample buffer (126 mM Tris–HCl (pH 6.8), 20% (v/v) glycerol, 4% (w/v) SDS, 0.02% (w/v) bromophenol blue), heated for 10 min at 95 °C and centrifuged for 1 min (4 °C; 16 100×g). Subfractionation of leaf material was performed as described previously (Schmied *et al.*, 2018). The resulting protein extracts were fractionated by 12% SDS-PAGE, blotted onto nitrocellulose membranes and probed with specific antibodies. Three blots were always performed for the protein samples and a representative image was selected for presentation.

### Thylakoid isolation and sucrose density gradient centrifugation

Thylakoids were isolated from leaves of 3-week-old Arabidopsis plants as previously described (Jarvi *et al.*, 2011) with one modification: the material was homogenized in a buffer containing 0.45 M sorbitol, 20 mM Tricine–KOH pH 8.4, 10 mM EDTA pH 8.0, and 10 mM NaHCO_3_. For sucrose density-gradient centrifugation, a 0.4–1.3 M sucrose gradient was prepared as described before (Trompelt *et al.*, 2014). Isolated thylakoids were adjusted to 0.8 mg Chl ml^−1^, mixed with 0.6% *n*-dodecyl β-d-maltoside, and loaded onto the gradient. Following ultracentrifugation for 16 h (4 °C; 134 470×g), the gradient was divided into 21 equal fractions. An aliquot of each fraction was mixed with an equal volume of 2× Laemmli sample buffer, heated for 10 min at 95 °C and centrifuged for 1 min (4 °C; 16 100×g). The supernatants were then used for immunoblotting analysis.

### Blue native polyacrylamide gel electrophoresis

Thylakoids were isolated as described above, adjusted to 0.8 mg ml^−1^ Chl and solubilized by using 1% *n*-dodecyl-β-maltoside for 30 min in the dark. After solubilization, the thylakoids were loaded onto a 4–12% blue native polyacrylamide gel and electrophoresis was conducted as described before (Jarvi *et al.*, 2011). Three independent experiments were performed for samples from Col-0, *ttp1*-2, *ttp1*-3, and TTP1-OE lines. No significant changes were observed for the samples of each line.

### Chlorophyll fluorescence and low temperature (77 K) chlorophyll fluorescence measurements

Leaves were adapted in the dark for 15 min before measurement. Afterwards, photosynthetic efficiency, quantum yield of PSII, and non-photochemical quenching were measured by a Pulse Amplitude Modulation (PAM)-imager according to manufacturer's instructions (Walz, Germany). Seedlings were homogenized in LHC-buffer (50 mM Tricine, 0.4 M sorbitol, pH 7.8), mixed 1:1 with 80% glycerin, transferred to a capillary, and carefully frozen in liquid nitrogen. Chlorophyll fluorescence was measured by fluorescence spectroscopy (F-7000, Hitachi, λ_ex_=440 nm; λ_em_=650–800 nm). The emission spectra were normalized to the emission at 720 nm.

### Bimolecular fluorescence complementation analysis

The coding sequences of interacting proteins were amplified from wild-type cDNA, and *attB* sites were attached prior to cloning via GATEWAY® into the pDEST-GW-VYNE (YFP^N^) and pDEST-GW-VYCE (YFP^C^) destination vectors, which contain the N-terminal and C-terminal halves of yellow fluorescent protein (YFP), respectively. The constructs (YFP^N^/YFP^C^) were then transformed into *Agrobacterium* (GV2260). For transfection, the *Agrobacterium* cultures were adjusted to OD_600nm_=0.6–1.0 with infiltration buffer (10 mM MES; 10 mM MgCl_2_; 100 μM acetosyringone). Equal volumes of the cultures expressing the interacting fusion proteins were mixed, and injected into *N. benthamiana* leaves. Transfected plants were kept for 2–3 d in darkness. Reconstituted YFP fluorescence was monitored with confocal laser-scan microscopy (Zeiss LSM 800; λ_ex_=514 nm; λ_em_=530–550 nm). Chloroplasts were localized by measuring chlorophyll autofluorescence (λ_ex_=488 nm; λ_em_=650–700 nm).

### Yeast two-hybrid assay

The coding sequences of the interacting proteins were amplified (without the transit peptide-encoding sequences) from wild-type cDNA. The amplified cDNA was either inserted via classical cloning into the pDHB1MCS2 vector (pDHB3.1; bait) or inserted via GATEWAY® cloning into the met25pXCgate vector (pNUB; prey). The bait and prey constructs were then transformed in L40ccuA and L40ccuα yeast cells, respectively. Transformation and mating were performed as previously described (Hackenberg and Grimm, 2012). Successfully mated yeast cells were selected on synthetic complete medium (4 g l^−1^ yeast nitrogen base without amino acids; 2% (w/v) glucose) without leucine and tryptophan. The selection of positive interactions was performed on synthetic complete medium without histidine, uracil, leucine, and tryptophan. To minimize false positive interactions, 20 mM 3-aminotriazole was added. The selection was performed for 3 d at 30 °C.

### Microscale thermophoresis assay

6×His–TTP1 (20 µM) was labeled using the RED-NHS Protein Labeling Kit (NanoTemper, Munich, Germany) according to the manufacturer’s instructions. Proteins were further diluted with phosphate-buffered saline after labeling. The degree of labeling was determined using UV-Vis spectrophotometry at 650 and 280 nm and was approximately 1.0. The binding reactions consisted of 200 nM labeled protein (constant) and decreasing amounts of the binding partner. Microscale thermophoresis (MST) measurements were performed in a Monolith NT.115 device using standard capillaries. Excitation and MST power were set to 100% and 40%, respectively. Data from independently pipetted measurements were analysed (MO.Affinity Analysis software v.2.1.3, NanoTemper Technologies) using the signal from an MST-on time of 1.5 s.

### Staining of reactive oxygen species

Hydrogen peroxide was stained with 3,3ʹ-diaminobenzidine (10 g l^−1^ in double-distilled water, pH 3.8) and superoxide anions were stained with nitro blue tetrazolium chloride (2 g l^−1^ in 50 mM sodium phosphate buffer, pH 7.5). In both cases, detached leaves were submerged in the staining solution and a low vacuum was applied for 2–3 min. The samples were then incubated overnight. Afterwards, the staining solution was replaced by 96% ethanol, and the samples were heated in a water bath (95 °C) to completely remove chlorophyll. Singlet oxygen staining was performed by using Singlet Oxygen Sensor Green (SOSG) according to the manufacturer’s instructions (Thermo Fisher Scientific, Germany). The signal was detected by confocal laser-scanning microscopy (Zeiss LSM 800; λ_ex_=504 nm; λ_em_=500–550 nm).

### Statistical analysis

Statistical analyses were performed with the RStudio Server (Version 1.3.1073, RStudio Team (2015), Boston, MA, USA; retrieved from http://www.rstudio.com/). Normality and variance homogeneity were tested with Shapiro and Levene tests, respectively. When samples were normally distributed and exhibited homogeneous variance, analysis of variance (ANOVA) was applied. When equal numbers of data points were considered, ANOVA was followed by the Tukey post-hoc test, and otherwise the Šidák post-hoc test was used. Compact letter display was based on α=0.05. When the samples were not normally distributed, the Kruskal–Wallis test was applied. Here the *P*-value was adjusted to the Bonferroni correction. The data visualization was done with the RStudio Server, using the ggplot2 package (v3.3.3) (Wickham, 2016).

## Results

### Identification of TTP1, a Pitt-like protein in Arabidopsis

Twenty-two TPR proteins were predicted to be localized in the plastids of Arabidopsis by various databases (Supplementary Table S1; TAIR, Chloroplast Function Database II, Uniprot; Bohne *et al.*, 2016). The prediction of the plastid localization was repeated with ChloroP (Supplementary Table S1). Candidates that lacked a clear transit peptide according to ChloroP were checked again using Cell-Ploc2.0 (Chou and Shen, 2008). Owing to the complex composition of the TPR motif, the alignment of homologous sequences suggested by database algorithms is not always reliable. For this reason, we used TPRpred (Karpenahalli *et al.*, 2007) to determine the numbers of TPR motifs and their lengths. This resulted in a 99% probability that 17 of the 22 selected candidates were indeed TPR proteins (Supplementary Table S1).

Sequence alignment of the 182-amino acid-long TPR domain of the cyanobacterial Pitt protein with those of homologous Arabidopsis proteins by BLAST analysis revealed six potential candidates with an overall sequence similarity of 41–49% and a sequence identity of 23–26% (Supplementary Fig. S1A). All of these TPR proteins contained a predicted N-terminal transit sequence enabling import of the precursor proteins into chloroplasts, and between five and seven TPR motifs. Of the six TPR protein variants finally selected, the TTP1 sequence encoded by *At1g78915* showed the highest similarity to the cyanobacterial Pitt protein due to the transmembrane domain, which is absent in all other variants. This suggests a close functional relationship between the two homologous proteins (Supplementary Fig. S1B, C).

Thus, we selected TTP1 as the most promising Pitt-like protein in Arabidopsis for further genetic and physiological analysis. Apart from the putative N-terminal transit peptide of the Arabidopsis homolog, both homologs comprise a predicted N-terminal transmembrane domain, followed by a potential linker sequence and the C-terminal TPR domain with the five canonical TPR motifs (Supplementary Fig. S1D). For comparison, the N-terminal segment of mature FLU possesses a transmembrane domain, in addition to three TPR motifs at the C-terminal end (Supplementary Fig. S1A).

### Impact of loss and over-production of TTP1 on the levels of tetrapyrrole biosynthesis enzymes and 5-aminolevulinic acid synthesis

To analyse the TTP1 function *in planta*, three individual *TTP1* T-DNA insertion lines were selected for further study. Genotyping and immunoblot analysis revealed that the homozygous *ttp1-2* mutant represents a *knockdown* line, because the T-DNA-insertion is located within the 5ʹ-untranslated region (Fig. 1B–G). The *ttp1-1* and *ttp1-3* alleles are knockout lines, as the T-DNA insertions were found in the first intron and the 15th exon, respectively (Fig. 1B–G). We generated a TTP1-specific antibody, and one immunoreactive protein band was detected in total wild-type leaf extracts upon fractionation by SDS-PAGE (Fig. 1G). Immune analysis verified the status of TTP1 knockdown and knockout lines showing deficiency and loss of TTP1, respectively, compared with wild type.

**Fig. 2.**
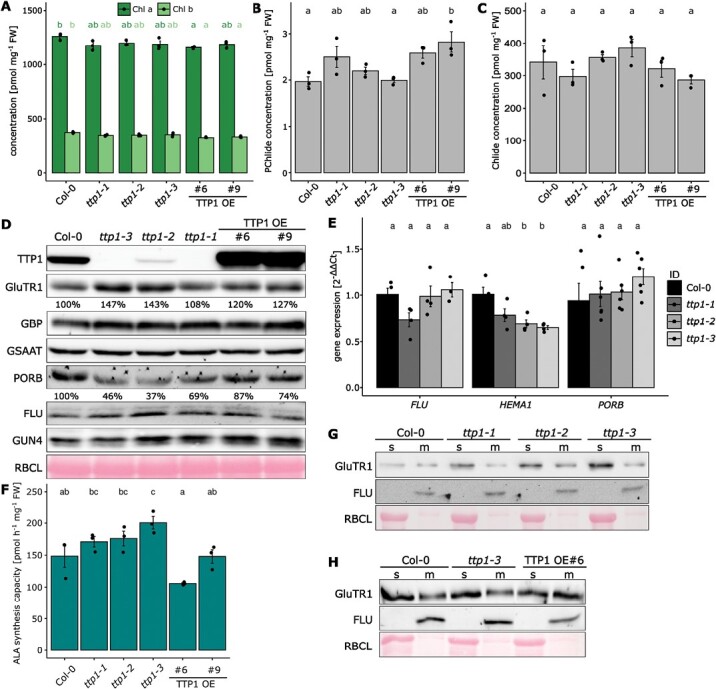
TTP1 affects the accumulation of GluTR1 and the ALA synthesis capacity. All analyses were performed in 3-week old plants grown under short day conditions (SD, 10 h light–14 h darkness, 120 µmol photons m^−2^ s^−1^, 21 °C, 60% humidity). (A–C) Chlorophyll *a* and *b* (Chl *a* and *b* (A)), protochlorophyllide (PChlide (B)), and chlorophyllide (Chlide (C)) concentrations relative to fresh weight (FW). Different lowercase letters above the bars indicate statistically significant differences (*P*<0.05). Significance was calculated with the Kruskal–Wallis test (Bonferroni post-hoc test; *n*=3). In (A) the different colors of the lowercase letters stand for the respective groups being compared. (D) The abundance of TBS proteins analysed by immunoblotting. (E) The relative gene expression levels of *FLU*, *HEMA1* (encoding GluTR1 protein), and *PORB* of the transgenic lines, determined in comparison with the wild type. Different lowercase letters above the bars indicate statistically significant differences (*P*<0.05). Significance was calculated with the Kruskal–Wallis test (Bonferroni post-hoc test; *n*≥4). (F) The ALA synthesis capacity in 4-week-old Arabidopsis seedlings measured for all investigated lines after incubation of leaves in levulinic acid for 4 h. Different lowercase letters above the bars indicate statistically significant differences (*P*<0.05). Significance was calculated with one-way ANOVA for analysis of ALA synthesis rate (Tukey post-hoc test; *n*=4). (G, H) The soluble (s) and membrane (m) localized fractions of GluTR1 analysed with specific antibodies. The abundance of FLU was used as a marker for the membranous fraction. The large subunit of RuBisCO (RBCL) was used as a loading control in (D, G, H). The relative intensities of the chemiluminescence signals of proteins were quantified with ImageJ.

We generated TTP1 overexpression (TTP1-OE) lines by introducing a p35S-TTP1 cassette into the *ttp1-3* mutant background. This increased the level of the *TTP1* transcripts by up to 6- to 8-fold and that of the TTP1 protein by 2- to 3-fold in representative lines (Fig. 1F, G). Compared with wild type, none of the three allelic *ttp1* mutants and neither of the two TTP1-OE lines grown under short-day (SD) conditions exhibited any obvious macroscopic changes (Fig. 1E). Leaf pigmentation of *ttp1* and TTP1-OE lines did not significantly change compared with wild-type plants, and similar levels of Chl *a* and *b* were measured (Fig. 2A). As the *Synechocystis pitt*^*−*^ strain accumulates less Chlide than the wild type (Rengstl *et al.*, 2011), we analysed the steady-state levels of TBS intermediates in the different transgenic lines grown under SD conditions and standard light intensities. The PChlide and Chlide contents of the three allelic *ttp1* mutants and the two TTP1-OE lines were not significantly modified in comparison with wild type (Fig. 2B, C). Because POR activity is strictly dependent on light, we also analysed the PChlide levels of all SD-grown transgenic lines after an extended dark period. No significant difference in PChlide content was observed in the *ttp1* and TTP1-OE lines relative to wild type (Supplementary Fig. S2A).

Additionally, since the Synechocystis *pitt* strain showed a strong effect on the photosynthetic performance and accumulation of photosynthetic complexes (Schottkowski *et al.*, 2009; Rengstl *et al.*, 2011), we examined similar parameters in *ttp1-2* and *ttp1-3* SD-grown seedlings (Supplementary Fig. S3). However, none of the investigated parameters nor the accumulation of photosynthetic protein complexes was significantly changed in comparison to the wild type.

We analysed the content of POR and other TBS enzymes in 4-week-old TTP1-deficient and overproducing lines and compared them with wild-type plants grown under SD conditions (Fig. 2D). The PORB content was reduced by 31–63% in all three allelic *ttp1* mutants, which could compromise POR activity. However, the steady-state levels of PChlide did not strikingly differ between leaf samples harvested from light- or dark-incubated seedlings (Fig. 2B; Supplementary Fig. S2A). Interestingly, the *ttp1* mutants contained slightly more GluTR than the wild type, and their GBP content also increased accordingly, while GSAAT and FLU contents remained wild type-like in all lines (Fig. 2D). The two selected TTP1-OE lines also tended to exhibit elevated levels of GluTR (Fig. 2D), while the amount of POR increased in comparison with that of the TTP1-deficient lines, but still lagged behind the amount of wild-type seedlings. The accumulation of other TBS proteins does not show a consistent pattern.

We further quantified the transcripts levels of *HEMA1* (encoding GluTR1), *FLU*, and *PORB* to ascertain whether or not they correspond to the relative contents of the encoded proteins. The *FLU* and *PORB* transcripts accumulated to levels similar to those in wild-type plants. Strikingly, *HEMA1* expression was reduced in *ttp1*-2 and *ttp1*-3 compared with wild type and did not correspond to the GluTR content (Fig. 2D, E). Therefore, we propose that post-translational modifications are likely to be responsible for the altered accumulation of TBS proteins in the *ttp1* mutants.

Because the content of the rate-determining enzyme GluTR was modified in the *ttp1* mutants, we assayed the ALA synthesis rate in all genotypes, grown under SD conditions. Among the three allelic *ttp1* mutants, *ttp1*-3 showed a significantly enhanced ALA synthesis rate compared with wild-type and TTP1-OE plants, whereas the ALA synthesis rates of the other two allelic mutants did not change significantly compared with the wild type (Fig. 2F). As the relative distribution of the GluTR content in stroma and membrane correlates with the ALA synthesis rate (Schmied *et al.*, 2018), we measured the levels of GluTR in these two subplastidal fractions of *ttp1* and wild-type leaves (Fig. 2G). The higher accumulation of GluTR in *ttp1* mutants is attributable to the elevated amounts of the protein in the stroma relative to the membrane-bound fraction. Compared with wild type, the TTP1-OE lines had non-significantly altered ALA synthesis capacity and only weakly increased amounts of GluTR at the membrane. (Fig. 2F, H). We therefore conclude that under SD conditions the deficiency or excess in TTP1 tends to have no significant impact on ALA synthesis rates at the time of measurement.

**Fig. 3.**
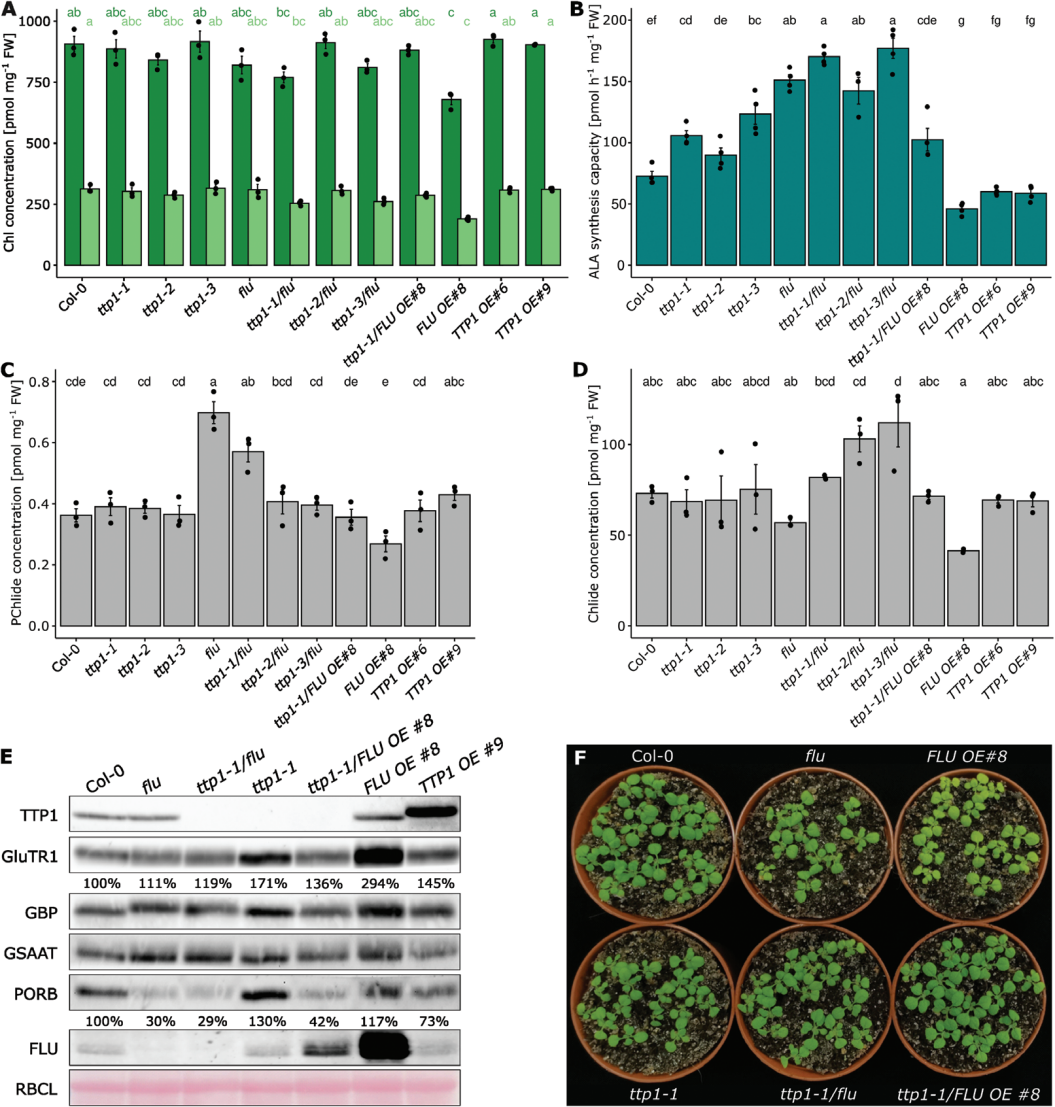
FLU and TTP1 act independently upon ALA synthesis. The indicated genotypes were grown under FL conditions (continuous light exposure with alternating 3 and 300 µmol photons m^−2^ s^−1^ at 15 min intervals) for 3 weeks. (A) The chlorophyll *a* and *b* (Chl *a* and *b* (A)), protochlorophyllide (PChlide (C)), and chlorophyllide (Chlide (D)) concentrations relative to fresh weight (FW). Different lowercase letters above the bars indicate statistically significant differences (*P*<0.05). The different colors of the lowercase letters stand for the respective groups being compared. Significance was calculated with the Kruskal–Wallis test (Bonferroni post-hoc test; *n*≥3). (B) The ALA synthesis capacity of the indicated TTP1 and FLU lines quantified after incubation of leaves with levulinic acid for 4 h. Significance was calculated with the Kruskal–Wallis test (Bonferroni post-hoc test, *P*<0.05, *n*≥4). (E) The abundance of the indicated TBS proteins analysed by immunoblotting. The large subunit of RuBisCO (RBCL) was used as a loading control. The quantification of the chemiluminescence was performed using ImageJ. (F) Phenotypes of the investigated TTP1 and FLU genotypes.

We carried out similar experiments with seedlings of the different genotypes grown under different growth conditions: continuous light (CL), long day (LD), SD, and fluctuating light (FL; continuous light exposure with alternating 3 and 300 µmol photons m^−2^ s^−1^ at 15 min intervals; Supplementary Fig. S4). The growth of transgenic lines with loss or excess of TTP1 resembled that of wild type. In anticipation of a greater challenge for the mutants lacking TTP1 or with excess TTP1, Chl, PChlide, and Chlide steady-state levels and ALA synthesis rates were monitored from plants grown under FL treatment (Fig. 3A–D). The Chl content was similar in all lines, consistent with the development of all seedlings (Fig. 3A,F). The two *ttp1* knockout lines showed a significantly higher ALA synthesis rate, which could be reversed in the TTP1-OE lines (Fig. 3B). These differences in ALA synthesis did not result in modified PChlide and Chlide levels (Fig. 3C, D). It is assumed that PChlide-converting POR and Chlide-utilizing chlorophyll synthase can get along with different amounts of their substrates without accumulation of these substrates when ALA synthesis is slightly increased or decreased (Fig. 3A–D), especially under conditions without a dark phase. Comparative immune analysis of several TBS proteins of SD- and FL-grown *ttp1-1* and TTP1-OE #9 lines revealed some modified GluTR and POR contents compared with wild type (Figs 2D, 3E). GluTR accumulated more in FL-grown *ttp1* than in wild type, which is consistent with the change in SD-grown *ttp1*-*1* relative to wild type. FL-grown *ttp1*-*1* accumulated more PORB than the wild type, and thus differed from the lower PORB amount of *ttp1*-*1* under SD conditions (Fig. 2D). In the FL-grown TTP1-OE #6 line, the content of GluTR was slightly elevated in comparison with the wild type, while the PORB content weakly decreased in contrast to wild type. Thus, the accumulation of GluTR and PORB in the TTP1-OE #6 line resembled the results from SD-grown plants.

Apart from the analysis of the impact of loss or excess of TTP1 on ALA and chlorophyll synthesis during light exposure, we also examined leaf samples of *ttp1* and TTP-OE lines after extended dark periods (Supplementary Fig. S2B, C). Samples form CL-grown leaves of all genotypes after an extended (16 h) dark period were harvested to establish whether the changes in TBS protein accumulation and ALA synthesis rate affect tetrapyrrole biosynthesis in darkness. Here, representative TTP1-deficient and -overaccumulating lines showed no correlation between the modified genotype of *TTP1* and the accumulation of PChlide (Supplementary Fig. S2B). Compared with the wild type, GluTR and POR levels of CL-grown lines after 16 h dark incubation were similar to in the FL-grown *ttp1*-*1* (Supplementary Fig. S2C).

### TTP1 affects the greening of etiolated Arabidopsis seedlings

**Fig. 4.**
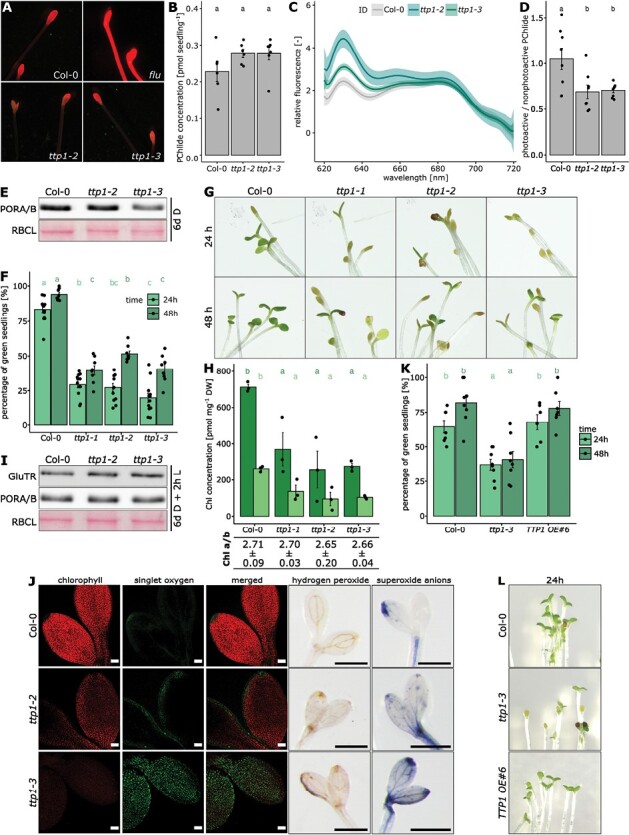
Analysis of *ttp1* mutants during etiolation and greening. Arabidopsis seeds were illuminated for 1 h with white light to induce germination and subsequently grown for 6 d in darkness. (A, B) PChlide accumulation visualized by fluorescence microscopy (A) or measured from seedling extracts by HPLC analysis (B). For the fluorescence microscopy, *flu* was used as positive control. (C) Low temperature (77 K) fluorescence measured in three biological and three technical replicates. The measurements were normalized to the fluorescence at 720 nm. The 99% confidence intervals were plotted. (D) Based on the emission spectra, the ratio of photoactive PChlide (emitting at 653 nm) versus non-photoreactive PChlide (emitting at 632 nm) was determined. Different lowercase letters above the bars in (B, D) indicate statistically significant differences (*P*<0.05). Statistical significance was calculated by the Kruskal–Wallis test (Bonferroni post-hoc test; *n*=8). (E) The abundance of PORA/B was determined after 6 d of darkness. RBCL was used as loading control. To study the greening process, 6-day-old dark-grown seedlings were subjected to continuous light (CL) conditions. (F) The percentage of green seedlings, relative to the total amount of germinated seedlings, measured 24 h and 48 h after transfer to CL. Different lowercase letters above the bars indicate statistically significant differences (*P*<0.05). The different colors of the lowercase letters stand for the respective groups being compared. Statistical significance was calculated by the Kruskal–Wallis test (Bonferroni post-hoc test; *n*≥8). (G) Phenotype of representative seedlings after 24 h and 48 h in CL. (H) After 24 h, the amount of Chl *a* and *b* and the Chl *a*/*b* ratio determined relative to the dry weight (DW). Different lowercase letters above the bars indicate statistically significant differences (*P*<0.05). The different colors of the lowercase letters stand for the respective groups being compared. Statistical significance was calculated by one-way ANOVA (Tukey post-hoc test; *n*=8). (I) The abundance of PORA/B and GluTR determined after 6 d of darkness, followed by 2 h of white light. RBCL was used as loading control. (J) The accumulation of ROS analysed in seedlings after 16 h of light incubation. Singlet oxygen was visualized by singlet oxygen sensor green (SOSG) using confocal microscopy. The autofluorescence of Chl was used to localize the signals. The accumulation of hydrogen peroxide and superoxide anions was analysed by using 3,3ʹ-diaminobenzidine and nitro blue tetrazolium chloride, respectively. (K) The percentage of green seedlings measured for the representative *TTP1* knockout (*ttp1-3*) and overexpression (*TTP1 OE #6*) lines. Different lowercase letters above the bars indicate statistically significant differences (*P*<0.05). The different colors of the lowercase letters stand for the respective groups being compared. The statistical test used was one-way ANOVA (Šidák post-hoc test; *n*≥6). (L) Phenotype of representative seedlings.

We analysed whether etiolated and greening *ttp1* seedlings at an earlier stage of development showed deficits in pigment synthesis or plastid development. PORA/B contents and PChlide accumulation were analysed in 6-day-old etiolated *ttp1* and wild-type seedlings (Fig. 4). For fluorescence microscopy of *ttp1* seedlings, the *flu* mutant was included as an additional control. PChlide fluorescence in *ttp1* was wild type-like and drastically differed from the high PChlide levels of *flu* (Fig. 4A). Quantitative PChlide determination revealed that etiolated wild-type and *ttp1* seedlings accumulated identical amounts of PChlide (Fig. 4B). Interestingly, 77 K fluorescence spectroscopy showed that *ttp1* has higher amounts of free versus bound PChlide compared with wild type (Fig. 4C, D). Therefore, it is possible that the absence of TTP1 destabilizes POR (Fig. 4E) or their oligomeric structure (Gabruk *et al.*, 2015), resulting in reduced accumulation of photoactive PChlide.

Six-day-old etiolated Arabidopsis seedlings were exposed to CL (90 µmol photons m^−2^ s^−1^) for 48 h. After only 24 h of light exposure, more than 80% of the wild-type seedlings opened green cotyledons, whereas approximately 70% of the *ttp1* seedlings still showed closed, yellowish cotyledons (Fig. 4F, G). The analysed chlorophyll content was consistent with these macroscopic observations, but the Chl *a*/*b* ratio did not change in comparison with the wild type (Fig. 4H) After 48 h in continuous light, almost all wild-type seedlings were greened, whereas the percentage of green seedlings increased to approximately 40–50% in all allelic *ttp1* mutants (Fig. 4F, G). Thus, the *ttp1* lines exhibited a delayed greening phase. The delay in greening of the ttp1 mutants did not correlate with the reduced content of GluTR1 and PORA/B, two enzymes in regulatory hot spots of chlorophyll synthesis (Fig. 4I), but only in response to TTP1 deficiency. Delayed greening of *ttp1* could be rescued by overexpression of TTP1 (Fig. 4K, L).

### TTP1 and candidates for protein interactions

**Fig. 5.**
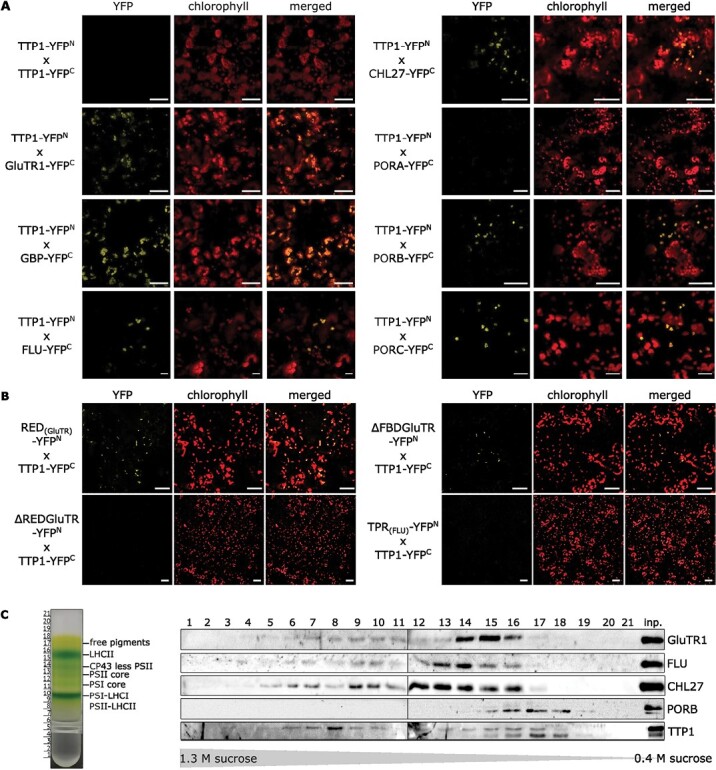
Interactions of TTP1 with enzymes of the TBS pathway *in planta*. (A, B) Bimolecular fluorescence complementation (BiFC) analysis of TTP1 with putatively interacting proteins. The indicated proteins were fused to either the N-terminal (YFP^N^) or C-terminal (YFP^C^) half of yellow fluorescent protein (YFP). Complementation was assessed by confocal microscopy 3 d after the infiltration of *N. benthamiana* leaves with recombinant *Agrobacterium tumefaciens*. Chl autofluorescence was used to prove the chloroplast localization of the interaction. Scale bar: 50 µm. (A) Images of BiFC assays after co-expression of TTP1–YFP^N^ with YFP^C^ fusion protein with TTP1, GluTR, GBP, FLU, CHL27, PORA, PORB, or PORC (from top left to bottom right). (B) Besides the different full-length proteins displayed in (A), the interactions between TTP1–YFP^C^ and the regulatory domain RED_(GluTR)_, the GluTR1 protein lacking either the RED (ΔREDGluTR) or the FLU-binding domain (ΔFBDGluTR), or the TPR domain of the FLU protein (TPR_(FLU)_) (always fused with the YFP^N^ peptide) were analysed (from top left to the bottom right). Scale bar: 50 µm. (C) Sucrose density-gradient analysis of isolated thylakoid membranes after 16 h of ultracentrifugation. The gradient was divided into 21 fractions of equal volume. Visible bands of the gradient were assigned according to Dall’Osto *et al.* (2010). The 21 fractions, as well as the isolated thylakoid membranes (inp.) of the sucrose density-gradient, were analysed for the abundance of TBS-related proteins by immunoblotting.

Interaction studies with TTP1, using bimolecular fluorescence complementation (BiFC) and split-ubiquitin-mediated yeast two-hybrid (Y2H), were initially focused on Arabidopsis POR, and subsequently extended to other enzymes and auxiliary factors of TBS. The BiFC assays revealed that TTP1 interacts with PORB and PORC, thus confirming the previously shown interaction of its cyanobacterial homolog Pitt with POR in *Synechocystis* (Schottkowski *et al.*, 2009). The BiFC assay also showed that TTP1 binds to GluTR, CHL27, GBP, and FLU (Fig. 5A). It is particular striking that this BiFC assay revealed interaction between the two TPR proteins, TTP1 and FLU. This interaction is remarkable, as it might be between TPR proteins with regulatory and assembly function. Interaction of TTP1 failed with itself and with PORA in these BiFC assays.

GluTR consists of several functional domains, of which the terminal ends are crucial for its interaction with other proteins (Zhao *et al.*, 2014; Apitz *et al.*, 2016; Richter *et al*., 2019). Therefore, two additional *HEMA1* constructs, which code for truncated GluTRs either lacking the regulatory domain (RED, previously called heme-binding domain), or the FLU-binding domain (FBD) were tested in this BiFC assay. Here, TTP1 did not interact with ΔREDGluTR, but bound to ΔFBDGluTR. As an additional verification, TTP1 was shown to bind to the isolated RED of GluTR (Fig. 5B), which implies that the membrane-bound TTP1 binds to the N-terminal part of GluTR and likely does not interfere with the FLU–GluTR interaction.

To verify the TTP1 interactions, a Y2H assay was performed (Supplementary Fig. S5). In contrast to the BiFC assays, this assay demonstrated the interaction of TTP1 with all three POR isoforms, including PORA. Moreover, an interaction of TTP1 with GluTR and CHL27, but not with FLU, was detected. This finding differs from results obtained by BiFC and MST assays (see below). It cannot be excluded that the detection of the interaction of TTP1 and FLU in the Y2H assay is hindered by their two hydrophobic domains. Alternatively, it is also possible that the interaction between TTP1 and FLU is mediated or enhanced by other interaction partners in the chloroplasts. Taken together, these results confirm direct TTP1 binding to POR, CHL27, GluTR, and GBP—each of which lacks any obvious transmembrane domain of its own.

To further analyse putative TTP1-containing complexes, we used sucrose density gradient ultracentrifugation to fractionate protein complexes solubilized from the plastid membranes of light-grown Arabidopsis wild-type seedlings, and characterized them by SDS-PAGE. Interestingly, two immunoreactive TTP1 bands were differentially distributed across the sucrose gradient after fractionation (Fig. 5C). The dominant TTP1_43kDa_ form was found in several fractions together with GluTR1, CHL27, and FLU. These four proteins also overlapped with TTP1_40kDa_ and PORB in fractions 15 and 16, which contained complexes of lower molecular mass (Fig. 5C). The two forms of TTP1 were detectable in membrane fractions of leaf samples, but not in total leaf extracts (Supplementary Fig. S1E; Fig. 1G). It remains unclear whether the two TTP1 variants are always present in the membrane and only detectable when the protein masses are very well separated in electrophoresis. The smaller variant of TTP1 could result from post-translational modification, such as proteolysis during purification of the thylakoid membranes. Nevertheless, we suggest that TTP1 serves as a scaffold for the assembly of homo- or hetero-oligomeric protein complexes involved in either ALA synthesis or the later stages of chlorophyll synthesis.

**Fig. 6.**
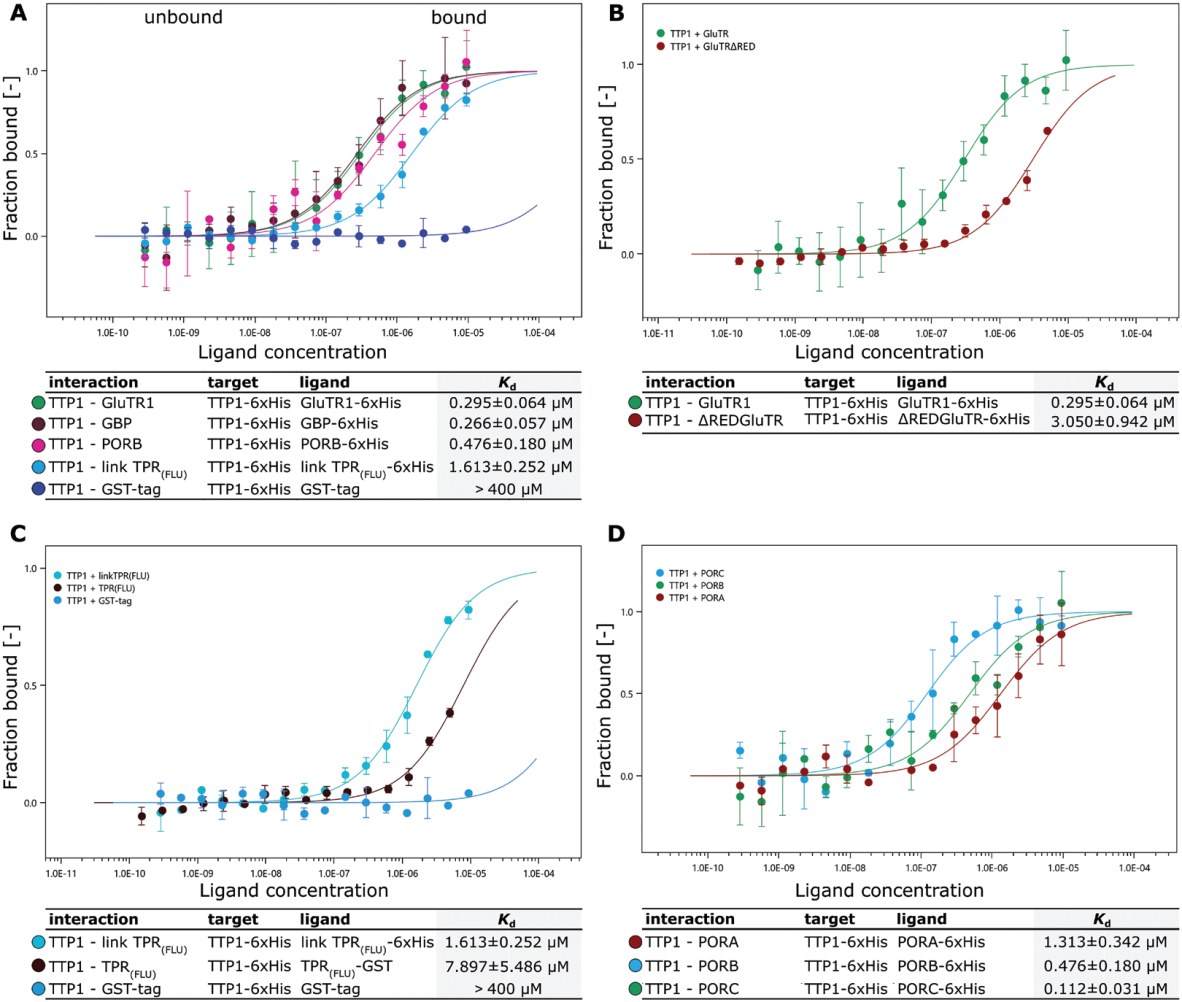
Interaction of TTP1 with different TBS proteins *in vitro*. Interactions between TTP1 and various TBS proteins were analysed by microscale thermophoresis and quantified in terms of their dissociation constants (*K*_d_ values). (A) The recombinant 6×His-tagged target protein TTP1 was titrated against different recombinant His-tagged proteins of tetrapyrrole biosynthesis in a temperature-induced concentration gradient. The glutathione *S*-transferase (GST) tag alone was used as control. (B) Analysis of the interaction of TTP1 with the full-length GluTR1, and with the GluTR1 protein lacking its regulatory domain (ΔREDGluTR). (C) Analysis of the interaction between TTP1 and the TPR domain of FLU, and with the linker+TPR domain of FLU. For TPR_(FLU)_ a GST-tagged version of the protein was used. (D) Analysis of the interactions between TTP1 and each of the three POR isoforms (PORA, B, and C). *K*_d_ fit is defined by the molecular interaction with a 1:1 stoichiometry according to the law of mass action. The statistics of the data shown are presented in Supplementary Fig. S6.

To quantify the relative affinities of TTP1 for its partners, we performed MST assays. The interaction of TTP1 with PORB, PORC, GBP, and GluTR revealed the highest affinity to these proteins among the tested proteins (Fig. 6A–D). For GluTR, GBP, and PORB/C, we measured dissociation constants below 1 µM, indicating a tight interaction under the tested conditions (Carlson *et al.*, 2008). Moreover, the data for the interactions of TTP1 with the different POR isoforms point to high binding affinities for PORC and PORB, but not for PORA. This assumption was supported by the lack of interaction between PORA and TTP1 detected in BiFC assays. With reference to Supplementary Fig. S5, it cannot be fully excluded, that TTP1 is able to directly interact with PORA under certain conditions.

TTP1 interaction assays with the complete FLU protein containing the transmembrane domain failed, owing to difficulties in expressing its hydrophobic segments in *E. coli*. Thus, we investigated the interaction affinity of TTP1 to two truncated FLU peptides expressing either the TPR domain (TPR_(FLU)_) or the linker domain followed by the TPR domain (link TPR_(FLU)_, Fig. 6C). While the TPR domain of FLU was previously shown to be essential for the interaction with both GluTR and POR (Zhang *et al.*, 2015), only a weak interaction to TTP1 could be detected, based on the *K*_d_ values. The interaction was intensified by the presence of the FLU linker domain. We suggest that the TTP1 and FLU interaction is further stabilized via the transmembrane domain of FLU, which could not be tested under these experimental conditions.

In summary, we conclude that TTP1 with its five TPR motifs not only interacts with POR at the plastid membrane, as it was presumed for the cyanobacterial Pitt protein, but in addition binds to GluTR, GBP, CHL27, and, with a small restriction, to FLU. These results point to a potential contribution of TTP1 to membrane-bound homo- and/or hetero-oligomeric protein complexes to TBS-associated proteins.

### The relationship between TTP1 and FLU

One of the potential TTP1-containing complexes that is associated with the plastid membranes, is the FLU-mediated GluTR-inactivation complex (Kauss *et al.*, 2012; Hou *et al.*, 2019). The essential components of this inactivation complex have been identified and its role in the PChlide-triggered suppression of ALA synthesis is accepted (Meskauskiene *et al.*, 2001; Goslings *et al.*, 2004). To explore the relationship between TTP1 and FLU, to compare the impact of these two proteins on TBS proteins involved in the inhibition of ALA synthesis, and to verify potential interdependencies between the two proteins, we analysed the single mutants side by side, together with representative overexpression lines of TTP1 and FLU. Additionally, we generated double mutants by crossing *ttp1* with *flu* and the FLU-OE line. Due to the cell-death phenotype of *flu* during illumination after a dark period, we applied continuous FL to all analysed lines. Immune-analyses confirmed the expected levels of TTP1 and FLU in the knockout mutants and the overexpression lines for FL growth (Fig. 3E). Apart from *ttp1*, also the FLU-OE line had a higher GluTR and POR content in FL-exposed leaf samples compared with wild type. The *flu* single and *ttp1-1/flu* double mutants accumulate less POR than wild type and point to the unstable FLU-containing GluTR inactivation complex, which operates not only in darkness but also under changing light conditions. It is worth noting that a drastically diminished FLU level was observed of the *ttp1-1*/FLU-OE line in comparison with the parental FLU-OE line (Fig. 3E).

The effects of a lack or excess of FLU or TTP1 were examined by determining ALA synthesis rates and the levels of PChlide and Chlide during FL growth. The data confirmed that *flu* and *ttp1* knockout mutants have higher rates of ALA synthesis than the wild type (Goslings *et al.*, 2004) (Fig. 3B), while the double mutants show no additive effects on ALA synthesis and achieve the synthesis rates of *flu*. Lower ALA synthesis rates were determined for the leaves of FLU-OE lines (Hou *et al.*, 2019), while TTP1-OE lines did not show significant changes when compared with wild type, which is consistent to the SD-grown samples. The *ttp1*/FLU-OE genotype exhibited a wild type-like capacity for ALA synthesis.

Steady-state levels of Chl and PChlide in the mutant genotypes may reflect effects on the GluTR inactivation complex and residual ALA synthesis. Regarding PChlide levels from light-exposed leaf samples, previous data on *flu* and FLU-OE lines (Goslings *et al.*, 2004; Hou *et al.*, 2019) were confirmed (Fig. 3C): *flu* accumulated more PChlide, whereas the FLU-OE line contained less than the wild type. Consistent with data from seedlings grown under SD conditions, PChlide concentrations at steady-state in the *ttp1-1* and TTP1-OE lines remained as in the wild type (Figs 2B, 3C). It was also found that the *ttp1-1*/*flu* double mutants were more characterized by wild-type levels of PChlide. Chlide steady-state values of all genotypes differed only insignificantly or not consistently.

### TTP1 and reactive oxygen species

Although TTP1 deficiency did not lead to any phenotypical change in growth or pigmentation under the investigated growth conditions (Figs 1E, 3F; Supplementary Fig. S3), ALA synthesis was slightly enhanced and the relative levels of proteins involved in ALA synthesis were perturbed in the FL-grown TTP1-deficient mutants compared with wild type (Fig. 3). It is likely that metabolic flows are modified, with minor changes in the steady-state levels of the photoreactive TBS intermediates, which could promote the generation of ROS. The formation of ROS was therefore analysed in leaves of 4-week-old seedlings of two TTP1-deficient lines. They indeed showed elevated accumulation of hydrogen peroxide and superoxide anions in comparison with wild type (Supplementary Fig. S7A). In agreement with these observations, the relative transcript levels of ROS marker genes were altered in comparison with wild-type plants. Especially the transcript levels of the singlet oxygen marker genes *BAP1* and *ATPase* were increased (Supplementary Fig. S7B), which indicates a cellular detoxification response towards singlet oxygen. To underline this observation, we investigated the accumulation of different ROS species in the cotyledons of greening *ttp1* seedlings. Indeed, the accumulation of singlet oxygen, hydrogen peroxide, and superoxide anions was increased compared with wild type (Fig. 4J). Hence, loss of TTP1 leads to the accumulation of ROS, which in turn triggers subcellular stress responses in *ttp1* mutants.

## Discussion

### TTP1 is an Arabidopsis homolog of the Pitt protein

TPR proteins generally mediate protein–protein interactions (D’Andrea and Regan, 2003). Their plastid-translocated representatives participate in multiple processes during plastid biogenesis, including TBS (Bohne *et al.*, 2016). Prompted by the characterization of the cyanobacterial Pitt protein (Schottkowski *et al.*, 2009), we considered a possible role for potential Pitt-homologous TPR proteins as regulatory or auxiliary factors involved in the post-translational control of TBS. We initially found six plastid-localized TPR proteins that exhibited a high degree of similarity with Pitt (Supplementary Table S1). Of these candidates, the membrane-bound TTP1 (At1g78915) was exclusively selected for further investigation because Pitt and TTP1 are most closely related in phylogenetic analyses (Supplementary Fig. S1B, C). TTP1 and Pitt share similarities in the composition of amino acid residues and the domain organization, which suggests that they may also have similar functions (Supplementary Fig. S1D). But most important, both proteins contain five canonical TPR motifs and an N-terminal transmembrane domain, which serves to anchor them to plastid membranes (Supplementary Fig. S1E) (Schottkowski *et al.*, 2009). The high similarity between the TPR motifs in Pitt and TTP1 suggests that they are true homologs. A phylogenetic search showed that transmembrane-containing homologs of TTP1 can be found in cyanobacteria, algae, monocots, and dicots.

Pitt stabilizes POR in *Synechocystis*, and it was suggested that it contributes to the subcellular localization of POR and the incorporation of chlorophyll into photosynthetic complexes (Schottkowski *et al.*, 2009; Rengstl *et al.*, 2011). TTP1 interacts with the membrane-associated proteins PORB and PORC, but may show only a weak interaction with PORA, the dominant isoform in etiolated seedlings (Figs 5A, D, 6A, D; Supplementary Fig. S5). Compared to *pitt*, TTP1 deficiency caused a slight decrease in PORB content under SD and FL conditions (Figs 2D, 3E) (Schottkowski *et al.*, 2009), but did not affect PChlide accumulation either in the light (Figs 2B and 3C) or in the darkness (Supplementary Fig. S2A, C). In contrast to the loss of Pitt, lack of TTP1 does not lead to decreased Chlide accumulation (Figs 2C, 3D). Moreover, unlike in *pitt*, the accumulation of chlorophyll and the activity and assembly of the photosynthetic complexes is not affected by the loss of TTP1 (Fig. 2A; Supplementary Fig. S3). The *ttp1* mutants did not differ in growth rate or pigmentation compared with wild type seedlings (Figs 1E, 2A, 3F; Supplementary Fig. S4).

A small malfunction in PChlide accumulation of etiolated *ttp1* mutants and a delayed greening of the yellow mutant seedlings were observed in comparative analysis with wild-type seedlings (Fig. 4), indicating the importance of TTP1 in the early stage of chloroplast biogenesis, when chlorophyll synthesis has to be greatly accelerated and coordinated with the assembly of the photosynthesis protein complexes. Compared with wild type, these physiological differences between etiolated and green *ttp1* seedlings indicate the necessity of TTP1 function in the early greening phase and underline the importance of a complex, multifactorial and balanced post-translational control of chlorophyll synthesis during early plant development. These observations of the de-etiolation mutant phenotype are consistent with enhanced expression of wild-type *TTP1*, especially in cotyledons and young leaf tissues (online available dataset of Klepikova *et al.*, 2016). Based on these results, it is proposed that the function of TTP1 is particularly required during chloroplast biogenesis at the greening stage of seedlings, while at later points in the development, TTP1 fulfills more of a supportive function for a smooth TBS.

### TTP1 contributes to the control of the glutamyl-tRNA reductase level bound to the plastid membrane and 5-aminolevulinic acid synthesis

While it was previously shown that Pitt interacts with POR, TTP1 does not exclusively bind to POR. It also interacts with CHL27, GluTR, and GBP, as demonstrated by a variety of methods—BiFC, Y2H, and MST assays (Figs 5, 6). FLU, another member of the TPR protein family, interacts directly with TTP1, but with a rather low affinity. Because the proof of interaction using Y2H failed (Supplementary Fig. S5), we assume that the interaction between TTP1 and FLU is rather indirect via other interaction partners. In addition to its impact on POR, the effects of loss or overproduction of TTP1 on the stability, activity, and subplastidal localization of GluTR were investigated. It is particularly striking that the GluTR content increases in the allelic *ttp1* knockout lines grown under SD and FL conditions (Figs 2D, 3E). The total amount of GluTR of the SD- and FL-grown TTP1-OE lines was also elevated (Figs 2D, G-H, 3E). Consequently, in addition to its binding to POR and other TBS proteins, analysis of mutants with loss or excess of TTP1 demonstrated a contribution of TTP1 to ALA synthesis, which always correlates with the assignment of GluTR to the plastidal stroma or thylakoid membrane. Based on our results the elevated GluTR content in *ttp1* correlates with a higher allocation of GluTR to the stroma (Fig. 2G, H).

In FL-grown seedlings, TTP1 deficiency is associated with enhanced ALA synthesis, while the elevated ALA synthesis of SD-grown *ttp1* was only insignificant (Figs 2F, 3B). However, this ALA synthesizing capacity correlates with higher levels of GluTR in the stroma relative to the membrane fraction (Fig. 2G). Conversely, excess of TTP1 correlates with a decrease in ALA synthesis in FL-grown transgenic lines (relative to wild type), but has little effect on ALA synthesis capacity during growth under SD (Figs 2F, 3B).

It is remarkable that both insufficiency and overproduction of TTP1 affect the stability of GluTR and POR under different growth condition and also ALA synthesis during continuous FL condition. These changes in *ttp1* seedlings are detectable, but remain often insignificant under analysed growth conditions (Supplementary Fig. S4), as several other GluTR-interacting proteins have to be considered, which contribute to the post-translational control of ALA synthesis in Arabidopsis (Wang *et al.*, 2022). These regulatory factors often have no obvious homologous or analogous counterparts in cyanobacteria. For example, formation of the GBP–GluTR complex is thought to inhibit proteolysis of GluTR by the soluble protease Clp, since the tight binding of GBP to the N-terminus of GluTR should ensure that the Clp selector proteins ClpS and ClpF have no access to the enzyme (Nishimura *et al.*, 2015; Apitz *et al.*, 2016). This mechanism would explain the elevated GluTR content in the *ttp1*-deficient lines, because the GBP content in the TTP1-deficient and -overproducing lines always resembles the changes in GluTR accumulation.

In addition to the role of GBP in protecting GluTR, we hypothesize that TTP1 helps to protect GluTR from degradation by establishing its attachment to the membrane. Thus, TTP1 could contribute to a GluTR-containing complex at the membrane. Independently of the dominant role of ALA synthesis in the soluble phase of plastids, TTP1 is assumed to stabilize GluTR at the membrane for the formation of the FLU-mediated GluTR inactivation complex. Alternatively, TTP1 might serve to maintain a back-up supply of GluTR. This last assumption is based on the concept of a dormant reserve of proteins that can be rapidly mobilized to meet sudden demands for ALA synthesis (Richter *et al.*, 2010). Such a steady-state pool of GluTR (which does not contribute to active catalysis under fluctuating environmental conditions) would always be available to appropriately adjust the tightly controlled synthesis of ALA and ensure an adequate flow of TBS metabolites.

### TTP1 and the stability of the FLU-mediated glutamyl-tRNA reductase inactivation complex

In addition to a role in maintaining adequate amounts of GluTR for the fine-tuning of ALA synthesis in the light and in the dark, we propose that TTP1 contributes to the stable association of POR and CHL27 at the membrane. Assuming a general safeguarding of POR, GluTR, and CHL27 by TTP1, its activity would most likely affect the membrane-localized GluTR-inactivation complex (Kauss *et al.*, 2012; Zhang *et al.*, 2015; Fang *et al.*, 2016). As revealed by sucrose density-gradient ultracentrifugation, two pools of TTP1 co-migrate with PORB, CHL27, GluTR1, and FLU (Fig. 5C). In this context, TTP1_43kDa_ could cooperatively act as a second TPR protein upon the same interaction partners to pre-prepare and stabilize the FLU-dependent GluTR inactivation complex. Thereby, the second variant, TTP1_40kDa_, seems to preferentially interact with POR at the plastid membrane. Thus, this interacting process could occur jointly or consecutively at the membrane. Moreover, since both TTP1 deficiency and overproduction in FL-grown seedlings correlate with higher and lower levels of ALA synthesis (relative to wild type), respectively, an auxiliary function of TTP1 in the assembly of the FLU-mediated GluTR inactivation complex is conceivable.

**Fig. 7.**
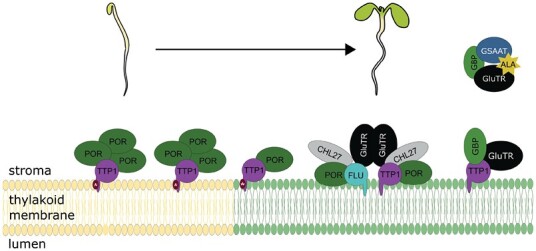
Working model of TTP1 function in Arabidopsis. It is proposed that in Arabidopsis seedlings TTP1 plays an indispensable role in the stabilization of POR during de-etiolation; larger complexes are not excluded. In contrast, in mature Arabidopsis tissue, at least three different membrane-associated complexes containing TTP1 are suggested, which may differ in the number of different proteins. One complex consists again of TTP1 and POR, in larger complexes or only as a heterodimer. This complex presumably contains a post-translationally modified version of TTP1, indicated by the red star. The second complex is made up of TTP1, GluTR1, and GBP, and likely serves in preparation for the interaction with other protein partners. Apart from TTP1, a final complex can contain GluTR1, FLU, CHL27, and POR and presumably function in stabilizing these proteins on the thylakoid membrane. This complex may be quite dynamic with varying numbers of interaction partners. It is suggested that these TTP1-containing complexes may feed into the FLU-containing GluTR inactivation complex, The two TPR proteins FLU and TTP1 likely do not interact. Besides its membrane-bound role, GluTR is bound in a soluble complex with GBP and GSAAT for ALA.

Increasing accumulation of PChlide in the dark as result of inactive POR normally triggers the FLU-mediated inactivation of GluTR (Richter *et al.*, 2010; Kauss *et al.*, 2012). We compared ALA synthesis rate and PChlide accumulation of *ttp1* and *flu*, their double mutants, as well as overexpression lines of both proteins to assess the TTP1 contribution to inactivation of ALA synthesis relative to the well-described function of FLU. The absence of TTP1 or FLU has a stimulating effect on ALA synthesis, but the effect of the absent FLU protein on ALA synthesis and PChlide accumulation is more pronounced. Remarkably, the *ttp1*/*flu* double mutant similarly shows impaired ALA synthesis and PChlide accumulation as previously determined in *flu* (Fig. 3B, C). As other light regimes and developmental stages of seedlings are used in our experiments in comparison with previous reports on *flu* (Meskauskiene *et al.*, 2001), the PChlide levels and ALA synthesis rates slightly differ. In consistency, the FL-grown TTP1 and FLU overexpression lines tend to show consistently lower ALA synthesis rates, but again only the FLU-OE #8 line has a significantly lower ALA synthesis rate and lower PChlide content compared with wild type (Fig. 3B, C). This comparison of data suggests a causal and correlated link of TTP1 to the FLU function on the inhibition of ALA synthesis. Either way, FLU retains the dominant effect on the GluTR inactivation complex in light and darkness compared with TTP1. We propose that TTP1 attaches GluTR to the thylakoid membrane and facilitates FLU-induced inhibition of ALA synthesis by (pre)binding soluble GluTR to the thylakoid membrane and then passing it on to the FLU-dependent inactivation complex (Fig. 7).

The lower affinity of TTP1 with the linker and TPR domain of FLU may coincide with the different roles of both TPR proteins. The dissociation constant in the MST assays implies at most only a moderate affinity between TTP1 and FLU, while the dissociation constants for TTP1’s interaction with GluTR, PORB, PORC, and GBP are lower, indicating substantially tighter binding (Fig. 6). Although we are aware of the artificial *in vitro* conditions for the MST measurements of the interactions of only two recombinant proteins at a time, we recall the previously determined dissociation constants of 42 nM for the GBP–GluTR interaction (Sinha *et al.*, 2022), 475 nM for the TPR domain of FLU with GluTR (and 2.3 µM with PORB) and between 14 and 200 nM for GluTR and PORB (Hou *et al.*, 2019). Most of these values suggest direct protein–protein interactions, which is less likely between TTP1 and FLU. But it is obvious that future studies are needed to verify the TTP1 contribution to complexes of multiple proteins, such as the GluTR inactivation complex.

### The role of TTP1 and Pitt in chlorophyll synthesis and photosynthesis

The common role of both homologous proteins Pitt and TTP1 is based on their localization to the thylakoid membrane via their respective N-terminal transmembrane domains. The previous analysis of the function of the Pitt protein concerned the effects of the interaction to POR (Schottkowski *et al.*, 2009). Based on the former and the current findings, it seems that both homologs might have similar and different functions at the thylakoid membrane. Although the lack of both proteins showed reduced amounts of POR, their impact on chlorophyll biosynthesis differs. Our studies have shown that TTP1 interacts with several TBS proteins. It may be useful to determine whether Pitt also interacts with other TBS enzymes. The involvement of Pitt in the subcompartmental localization of the biogenesis of chlorophyll-binding proteins and the assembly of photosynthetic protein complexes has been suggested (Schottkowski *et al.*, 2009), although it remains to be clarified whether the observation of impaired assembly of photosynthetic protein complexes is a direct consequence of the absence of Pitt. No changes in abundance and quality of photosynthetic complexes could be detected in Arabidopsis lines with lack of TTP1 (Supplementary Fig. S3).

A reason for additional TBS interactors of TTP1 may reflect the increasing complexity of the control of chlorophyll synthesis in higher plants relative to cyanobacteria. Apart from POR, *Synechocystis* still additionally operates with the dark-dependent POR, which synthesizes Chlide in darkness (Fujita, 1996). Since chlorophyll biosynthesis in Arabidopsis is strictly light-dependent, the functionality of TTP1 might be different and therefore the interaction of TTP1 with additional TBS proteins might be explained. An orchestrated control of light-dependent synthesis requires the simultaneous control of enzymes of ALA synthesis, POR, and enzymes of late chlorophyll synthesis. TTP1 participates in the subplastidal organization of TBS. Thus, the additional demands made by exclusively light-dependent chlorophyll synthesis have likely led to an extended function of the eukaryotic Pitt homolog TTP1.

### TTP1 is involved in the suppression of reactive oxygen species production

Notably, the loss of TTP1 does not have comparably serious effects on TBS to those described for other regulatory factors, like FLU (Meskauskiene *et al.*, 2001), GBP (Czarnecki *et al.*, 2011), LIL3 (Hey *et al.*, 2017), YCF54 (Herbst *et al.*, 2018), or PCD8 (Geng *et al.*, 2023). Nevertheless, any inefficient flow of TBS intermediates, as well as (partially) unbound TBS intermediates, leads to oxidative stress (Tripathy and Oelmuller, 2012). Indeed, loss of TTP1 leads to increased levels of singlet oxygen, superoxide anions, and hydrogen peroxide in etiolated seedlings 16 h after light exposure (Fig. 4J), and etiolated *ttp1* seedlings contain slightly more photosensitive, non-enzyme-bound PChlide than wild-type seedlings (Fig. 4C, D) Moreover superoxide anions and hydrogen peroxide accumulate in 4-week-old Arabidopsis plants grown under SD conditions, and increased expression levels of several ROS marker genes were observed (Supplementary Fig. S7A, B). Therefore, we do not exclude that the lack of TTP1 potentially leads to a deregulated flux of accumulating TBS intermediates. It should also be considered that the absence of TTP1 could lead to unstable or only loosely associated TBS protein complexes, which may then mark the beginning of a less protected transfer of intermediates to the next enzymatic step. The reduced amounts of PORB in green (Fig. 2D) or PORA/B in etiolated *ttp1* seedlings compared with wild type could transiently increase levels of unbound intermediates, which can generate ROS upon illumination.

### Conclusion

In summary, we conclude that TTP1 serves as an auxiliary factor that interacts with GluTR, POR, and CHL27 at the membrane. We propose that the role of TTP1 is based on its transmembrane domain, which allows it to anchor GluTR, POR, and CHL27 to the plastid membrane (Fig. 7). These enzymes are active at prominent metabolic checkpoints in the post-translational control of TBS, with the potential need to interact with several other regulatory and auxiliary factors (Wang *et al.*, 2022). One essential regulatory step that benefits from TTP1 is the FLU-dependent suppression of ALA synthesis. The two TPR proteins TTP1 and FLU have clearly distinguishable functions. Further investigations will elucidate the molecular details of the interactions of TTP with GluTR, POR, and CHL27 as a prerequisite for the formation of protein complexes either for the stabilized activation of these enzymes in ALA synthesis and Chlide formation, or for the GluTR inactivation complex.

## Supplementary data

Supplementary data are available at *JXB* online.

Fig. S1. Identification and analysis of the putative Pitt homolog TTP1 *in silico*.

Fig. S2. PChlide accumulation after prolonged dark phases.

Fig. S3. Abundance and activity of the photosynthetic complexes in two *ttp1* lines

Fig. S4. Phenotype of the *ttp1-3* knockout mutant and two overexpression lines under different light conditions.

Fig. S5. Split-ubiquitin analysis.

Fig. S6. Statistics of the data shown in Fig. 6.

Fig. S7. Detection of reactive oxygen species in *ttp1* knockout and knockdown lines

Table S1. List of chloroplast-localized TPR proteins and comparison of the identified TPR motifs with the TPR motif in Pitt.

Table S2. List of the oligonucleotides used.

erad491_suppl_Supplementary_Tables_S1-S2_Figures_S1-S7

## Data Availability

All relevant biological materials (Arabidopsis mutants, gene constructs for subcloning and transformation) are available from the corresponding author upon request. Supporting data are available in the Supplementary data published online.

## Abbreviations

ALA: 5-aminolevulinic acid
BiFC: bimolecular fluorescence complementation
Chl: chlorophyll
Chlide: chlorophyllide
CL: continuous light
Clp: caseinolytic protease
FL: fluctuating light
FLU: FLUORESCENCE IN BLUE LIGHT
GluTR: glutamyl-tRNA reductase
GBP: GluTR-binding protein
GSAAT: glutamate-1-semialdehyde aminotransferase
MST: microscale thermophoresis
PChlide: protochlorophyllide
Pitt: POR-interacting TPR protein
POR: protochlorophyllide oxidoreductase
RED: regulatory domain
ROS: reactive oxygen species
SD: short day
TBS: tetrapyrrole biosynthesis
TPR: tetratricopeptide-repeat
TTP1: tetrapyrrole biosynthesis-regulating tetratricopeptide-repeat protein1
Y2H: yeast two-hybrid
YFP: yellow fluorescent protein
