# Supplementary material for: A novel tetratricopeptide-repeat protein, TTP1, forms complexes with glutamyl-tRNA reductase and protochlorophyllide oxidoreductase during tetrapyrrole biosynthesis
Source: J Exp Bot. 2023 Dec 9;75(7):2027–45. doi: 10.1093/jxb/erad491 (PMC10967246; doi:10.1093/jxb/erad491)
Supplement: erad491_suppl_Supplementary_Tables_S1-S2_Figures_S1-S7 [file erad491_suppl_supplementary_tables_s1-s2_figures_s1-s7.pdf]

A

ID	name	%similarity	%identity	TPR motifs	TM
At1g78915	TTP1	45	26	5	yes
At3g18420	SG1	49	26	5	no
At3g37400	--	46	23	7	no
At2g39470	--	49	25	6	no
At5g02590	--	45	26	6	no
At3g53560	--	41	25	5	no
At3g14110	FLU	28	16	3	yes

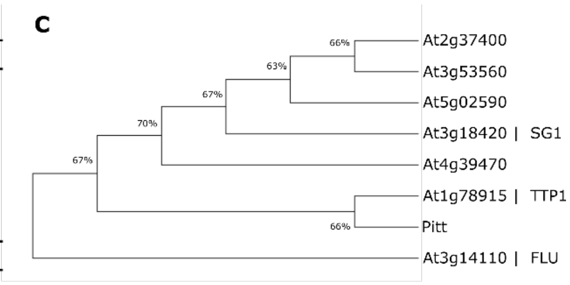

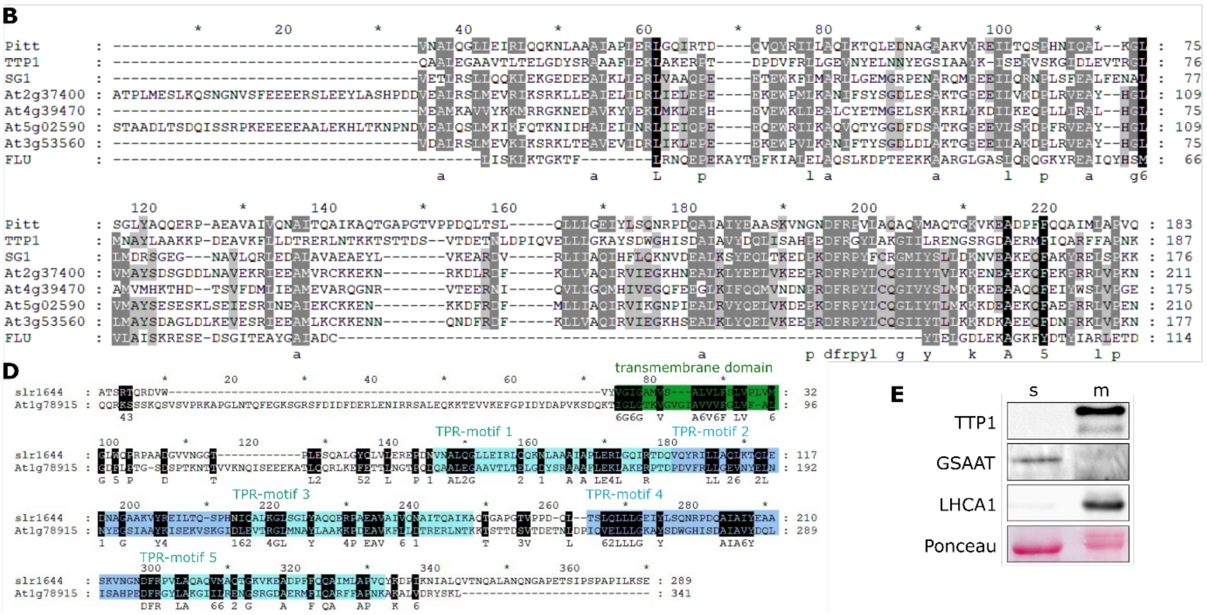

Fig. S1: Identification and analysis of the putative Pitt homolog TTP1 *in silico*. (A) In total, six TPR proteins from *A. thaliana* (in addition to the FLU protein) were analyzed. The levels of sequence similarity and identity of the identified TPR domain between the chosen TPR proteins and Pitt from *Synechocystis sp.* strain PCC 6803 were calculated with the Needleman-Wunsch Global Alignment by Basic Local Alignment Search Tool (BLAST). Moreover, the number of TPR motifs calculated with TPRpred (Version 11.0) and the presence of a transmembrane domain (TM) as defined by the TMHMM Server (v. 2.0) is given. (B) Multiple sequence alignment of the TPR domains of potential Pitt homologs in *A. thaliana*. The alignment was performed by Multiple Sequence Comparison by Log-Expectation (MUSCLE) (Edgar, 2004) and visualized with GeneDoc (Vers. 2.7.000). Conserved amino acids are highlighted in black or grey, depending on the number of TPR proteins showing this residue. (C) The coding sequences of the identified putative candidate Pitt homologs, together with Pitt and FLU, were used to create a phylogenetic tree based on a sequence alignment calculated by MUSCLE. The tree was constructed by the maximum-likelihood method and the Jones-Taylor-Thornton (JTT) matrix (Jones *et al.*, 1992) , and was visualized by MEGA-X (Version 10.0.5, (Kumar *et al.*, 2018)). The probability of the branching is given in percent. (D) Alignment of the protein sequences of Pitt (slr1644) and TTP1 (At1g78915) without the predicted chloroplast transit peptide of TTP1. The structural domains and motifs were predicted using TMHMM Server (v. 2.0) and TPRpred (Version 11.0). The alignment was performed by MUSCLE and visualized with GeneDoc (Vers. 2.7.000). Conserved amino acids are highlighted in black. Letters under the alignment represent identical amino acids, numbers represent the type of amino acid: 1 – negatively charged, 2 – positively charged, 3 – amidic, 4 – hydroxylic, 5 – aliphatic, 6 – large, 7 – small,

8 – sulphurous. (E) For subcellular localization of TTP1, isolated chloroplasts were fractionated into soluble (s) and membrane (m) fractions, which were probed with appropriate antibodies. GSAAT and LHCA1 were used as markers for the soluble and the membrane fractions, respectively.

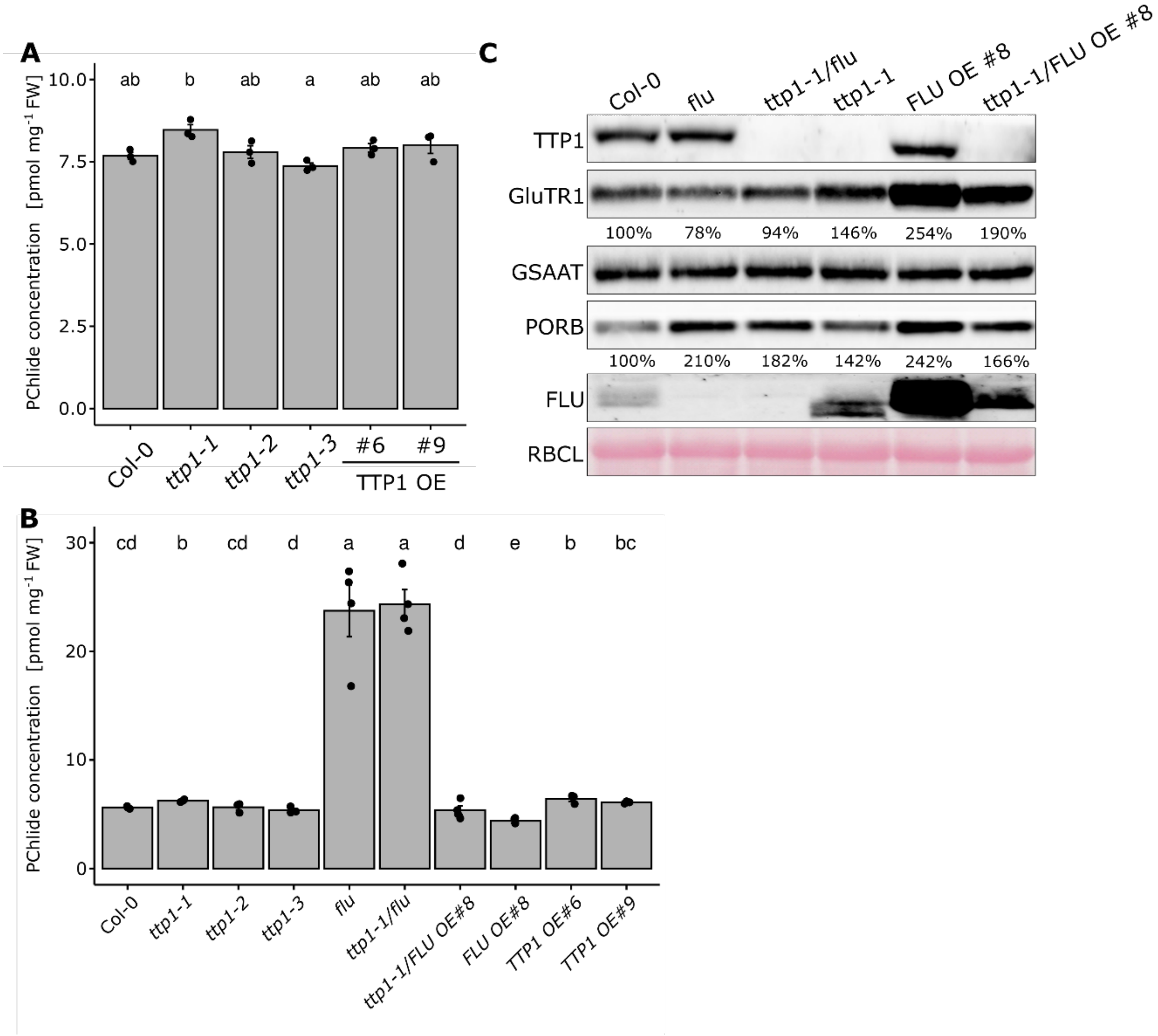

Fig. S2: PChlide accumulation after prolonged dark phases. The accumulation of PChlide was measured in 3-week-old plants grown under short-day conditions (A; SD, 10 h light/14 h dark, 120 µmoles photon m^{-2} s^{-1}, 21 °C, 60 % humidity) or continuous light (B; 24 h light, 120 µmoles photon m^{-2} s^{-1}, 21 °C, 60 % humidity) followed by 16 h of dark incubation. The PChlide concentrations are quantified relative to fresh weight (FW) in (A) and (B). Statistical significance was calculated by one-way ANOVA (Tukey post-hoc test, $p < 0.05$, n = 3). (B) The accumulation of PChlide was measured in CL-grown seedlings which were subjected to an extended dark phase of 16 h. Statistical significance was calculated by Kruskal Wallis Test (Bonferroni post-hoc test, $p < 0.05$, $n \geq 3$). (C) Three-week-old seedlings, grown under CL were subjected to 16 h of dark incubation. The abundance of TBS proteins was analyzed by immunoblotting and RBCL was used as loading control. The relative intensities of the chemiluminescent signals of GluTR1 and PORB were quantified with Image J.

A

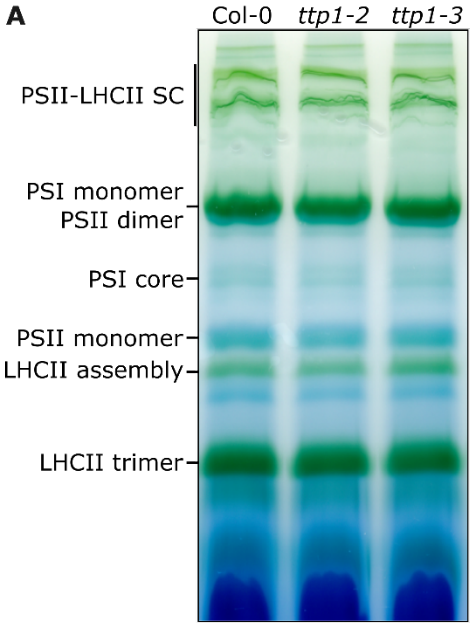

B

parameter	Col-0	*ttp1-2*	*ttp1-3*
Fv/Fm	0.840 ± 0.004	0.837 ± 0.009	0.836 ± 0.003
φPSII	0.661 ± 0.034	0.693 ± 0.039	0.714 ± 0.013
NPQ	0.366 ± 0.023	0.263 ± 0.145	0.282 ± 0.024

C

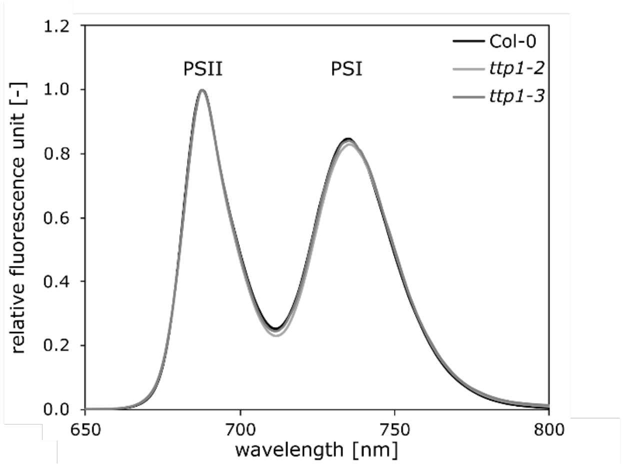

Fig. S3: Abundance and activity of the photosynthetic complexes in two *ttp1* lines. (A) Thylakoids of five-week-old Arabidopsis seedlings were isolated, solubilized with 1 % of n-dodecyl-beta-maltoside and separated on a 4- 12 % blue native polyacrylamide gel. For each sample an equivalent amount of Chl was loaded. (B) Photosynthetic parameters were analyzed by Pulse-amplitude modulation after a 15 min dark adaptation of the used leaves. (C) 77K chlorophyll florescence was measured in three biological and three technical replicates. The measurements were normalized to the fluorescence at 720 nm and the averaged values were plotted.

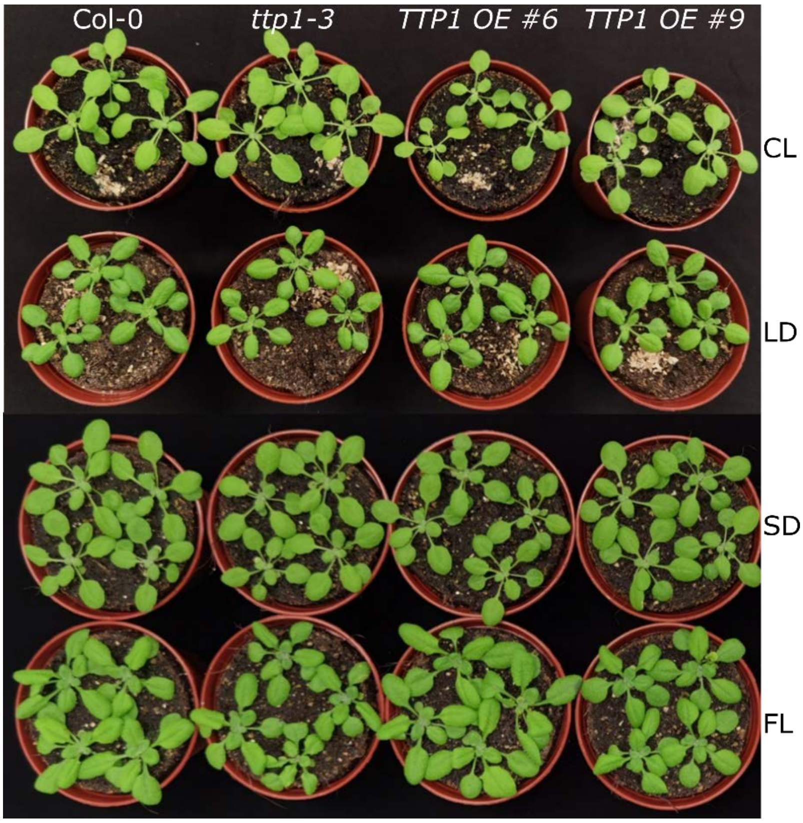

Fig. S4: Phenotype of the *ttp1-3 knockout* mutant and two overexpression lines under different light conditions. All investigated lines were grown for three weeks under the indicated light conditions, including continuous light (CL), long day (LD), short day (SD) and fluctuating light (FL).

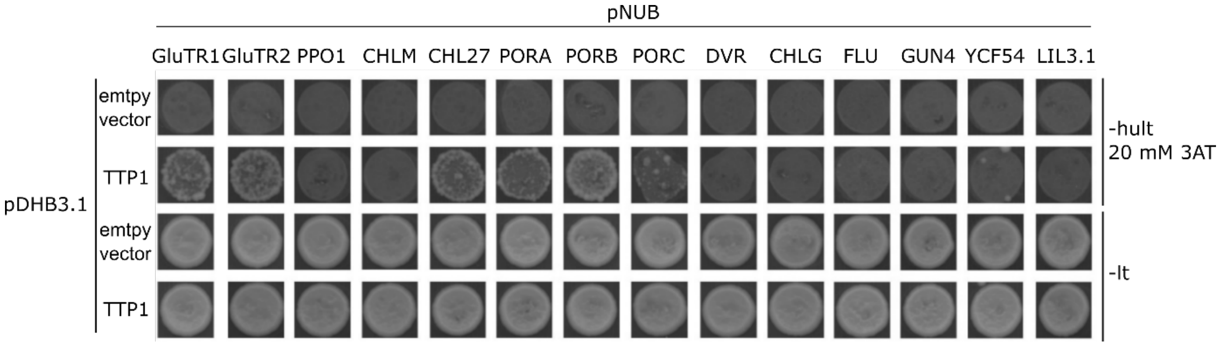

Fig. S5: Split-ubiquitin analysis. The indicated proteins were fused to the N-terminal (pNUB) or C-terminal (pDHB3.1) half of ubiquitin, and the different TBS enzymes and auxiliary factors were analyzed for interaction with TTP1. Successful co-transformation of both constructs was confirmed by growth on Leu- and Trp-deficient (-lt) media. In the case of an interaction, cells were able to grow on minimal media without His, Ura, Leu and Trp (-hult). To reduce the incidence of false negative results, 20 mM 3-aminotriazole (3AT) was added. The empty pDHB3.1 vector was used as the negative control.

NanoTemper Technologies

MO.Affinity Analysis v2.3

Origin of exported data	
Project Title:	
Comment:	
Project File Path:	J:\7 MST\2021-09-14 TTP1-his\14-11-2022.nta
Analysis-Set Name:	TTP1
Exported from:	Compare Results (Chart)
Exported on:	2023-11-19 16:18:31.165

TTP1 + GluTR

Sample Information	
Merge-Set Name:	TTP1 + GluTR
Date of Measurement:	2021-09-14 11:59:38.936 → 2021-10-21 13:05:53.199
Capillary Type:	Monolith NT.115 Capillary
Target:	TTP1-his
TargetConcentration:	0.00000002
Ligand:	GluTR-his

Measurement Settings	
MST-Power:	Medium
Excitation-Power:	40%
Excitation type:	Nano - RED
Thermostat Setpoint:	(disabled)

Analysis Settings	
Type of Analysis:	MST
Evaluation strategy:	Manual
Cold Region Start:	-1s
Cold Region End:	0s
Hot Region Start:	0.5s
Hot Region End:	1.5s

Fit Results (Kd)	
Fit Model:	Kd
Bound	947.3069895
Unbound	954.1749866
Kd	2.95163E-07
TargetConc	0.00000002 [illegible]
Standard Deviation:	0.452277701
Kd Confidence:	±6.41563E-08

Dose Response Curve		
Dose	Response	Err-Values
2.86865E-10	-0.08464544	0.1030081
5.7373E-10	0.037036253	0.1370727
1.14746E-09	0.014702702	0.0704465
2.29492E-09	-0.040900291	0.1553269
4.58984E-09	-0.013772474	0.1582633
9.17969E-09	0.074612385	0.1948764
1.83594E-08	0.016570566	0.1147891
3.67188E-08	0.265849453	0.1868291
7.34375E-08	0.170184595	0.1690331
1.46875E-07	0.310193303	0.0808468
2.9375E-07	0.490038196	0.1052113
5.875E-07	0.601935283	0.0789403
0.000001175	0.83322758	0.1066289
0.00000235	0.915054642	0.0843193
0.0000047	0.86235028	0.0697052
0.0000094	1.023016595	0.158914

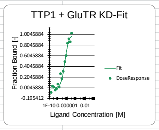

TTP1 + GBP

Sample Information	
Merge-Set Name:	TTP1 + GBP
Date of Measurement:	2021-09-14 14:10:29.197 → 2021-10-21 12:29:57.67
Capillary Type:	Monolith NT.115 Capillary
Target:	TTP1-his
TargetConcentration:	0.00000002
Ligand:	GBP-his

Measurement Settings	
MST-Power:	Medium
Excitation-Power:	40%
Excitation type:	Nano - RED
Thermostat Setpoint:	(disabled)

Analysis Settings	
Type of Analysis:	MST
Evaluation strategy:	Manual
Cold Region Start:	-1s
Cold Region End:	0s
Hot Region Start:	0.5s
Hot Region End:	1.5s

Fit Results (Kd)	
Fit Model:	Kd
Bound	949.2348096
Unbound	958.1521607
Kd	2.66537E-07
TargetConc	0.00000002 [illegible]
Standard Deviation:	0.571819654
Kd Confidence:	±5.66715E-08

Dose Response Curve		
Dose	Response	Err-Values
2.86865E-10	-0.054348432	0.1318795
5.7373E-10	-0.129796181	0.1992944
1.14746E-09	0.015836043	0.0496264
2.29492E-09	0.033621187	0.0376175
4.58984E-09	0.104114995	0.0712554
9.17969E-09	0.058865848	0.0100823
1.83594E-08	0.094157923	0.1071689
3.67188E-08	0.137009832	0.1484389
7.34375E-08	0.224206843	0.1681497
1.46875E-07	0.33280443	0.082326
2.9375E-07	0.427949758	0.1213613
5.875E-07	0.698700612	0.1326094
0.000001175	0.897251628	0.1629306
0.0000047	0.953295917	0.2459872
0.0000094	0.923439859	0.0963781

Dose Response Curve (Outlier and Hidden)		
Dose	Response	Err-Values
0.00000235	0.751282842	0.3691829

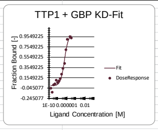

TTP1 + PORB

Sample Information	
Merge-Set Name:	TTP1 + PORB
Date of Measurement:	2021-09-14 12:47:56.851 → 2021-09-14 15:22:50.677
Capillary Type:	Monolith NT.115 Capillary
Target:	TTP1-his
TargetConcentration:	0.00000002
Ligand:	PORB-his

Measurement Settings	
MST-Power:	Medium
Excitation-Power:	40%
Excitation type:	Nano - RED
Thermostat Setpoint:	(disabled)

Analysis Settings	
Type of Analysis:	MST
Evaluation strategy:	Manual
Cold Region Start:	-1s
Cold Region End:	0s
Hot Region Start:	0.5s
Hot Region End:	1.5s

Fit Results (Kd)	
Fit Model:	Kd
Bound	947.863287
Unbound	953.3091313
Kd	4.75972E-07
TargetConc	0.00000002 [illegible]
Standard Deviation:	0.594811254
Kd Confidence:	±1.79799E-07

Dose Response Curve		
Dose	Response	Err-Values
2.86865E-10	-0.127058166	0.1748536
5.7373E-10	-0.158399224	0.152373
1.14746E-09	0.015664668	0.2551158
2.29492E-09	0.102849331	0.0069679
4.58984E-09	-0.068292634	0.0647296
9.17969E-09	-0.010816521	0.0603499
1.83594E-08	0.162764222	0.0830044
3.67188E-08	0.265084147	0.0784019
7.34375E-08	0.091058007	0.1782882
1.46875E-07	0.250408605	0.024418
2.9375E-07	0.409659738	0.0335609
5.875E-07	0.595077421	0.1011895
0.000001175	0.552887195	0.062667
0.00000235	0.784317119	0.0630126
0.0000047	0.904928505	0.0697307
0.0000094	1.052939671	0.1934367

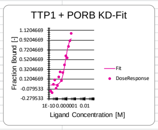

TTP1 + linkTPR(FLU)

Sample Information	
Merge-Set Name:	TTP1 + linkTPR(FLU)
Date of Measurement:	2021-09-14 13:50:11.351 → 2021-09-14 15:59:02.299
Capillary Type:	Monolith NT.115 Capillary
Target:	TTP1-his
TargetConcentration:	0.00000002
Ligand:	linkTPR(FLU)-his

Measurement Settings	
MST-Power:	Medium
Excitation-Power:	40%
Excitation type:	Nano - RED
Thermostat Setpoint:	(disabled)

Analysis Settings	
Type of Analysis:	MST
Evaluation strategy:	Manual
Cold Region Start:	-1s
Cold Region End:	0s
Hot Region Start:	0.5s
Hot Region End:	1.5s

Fit Results (Kd)	
Fit Model:	Kd
Bound	942.7823804
Unbound	957.0926479
Kd	1.61332E-06
TargetConc	0.00000002 [illegible]
Standard Deviation:	0.497033673
Kd Confidence:	±2.51942E-07

Dose Response Curve		
Dose	Response	Err-Values
2.86865E-10	-0.042768192	0.0814818
5.7373E-10	-0.031469591	0.0039528
1.14746E-09	0.053163784	0
2.29492E-09	0.008238834	0.0224302
4.58984E-09	-0.004260207	0.0015705
9.17969E-09	-0.025551416	0.0102486
1.83594E-08	0.012745303	0.044596
3.67188E-08	0.054736543	0.0286046
7.34375E-08	0.052083602	0.0057841
1.46875E-07	0.118186566	0.0313964
2.9375E-07	0.157194854	0.0353633
5.875E-07	0.240677786	0.0688345
0.000001175	0.371958421	0.077501
0.00000235	0.632275915	0.0013593
0.0000047	0.776689667	0.0161499
0.0000094	0.822225201	0.0377151

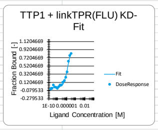

TTP1 + GST-tag

Sample Information	
Merge-Set Name:	TTP1 + GST-tag
Date of Measurement:	2021-09-14 16:47:09.596 → 2021-09-14 17:16:05.898
Capillary Type:	Monolith NT.115 Capillary
Target:	TTP1-his
TargetConcentration:	0.00000002
Ligand:	GST-tag

Measurement Settings	
MST-Power:	Medium
Excitation-Power:	40%
Excitation type:	Nano - RED
Thermostat Setpoint:	(disabled)

Analysis Settings	
Type of Analysis:	MST
Evaluation strategy:	Manual
Cold Region Start:	-1s
Cold Region End:	0s
Hot Region Start:	0.5s
Hot Region End:	1.5s

Fit Results (Kd)	
Fit Model:	Kd
Bound	949.4716543
Unbound	913.0791351
Kd	0.0004 [illegible]
TargetConc	4E-14
Standard Deviation:	1.146055325
Kd Confidence:	n/a

Dose Response Curve		
Dose	Response	Err-Values
2.86865E-10	0.037795486	0.0457248
5.7373E-10	0.020608065	0.0091712
1.14746E-09	0.01551668	0.0393934
2.29492E-09	-0.011360209	0.0611748
4.58984E-09	0.037865687	0.0261589
9.17969E-09	0.036186797	0.0176879
1.83594E-08	-0.012171471	0.0615595
3.67188E-08	-0.047988769	0.0243161
7.34375E-08	-0.03331521	0.0067545
1.46875E-07	0.023881239	0.0101713
2.9375E-07	0.000109952	0.0636383
5.875E-07	-0.021221645	0.0215364
0.000001175	-0.044536088	0.0046003
0.00000235	0.017878057	0.0873373
0.0000047	-0.012787475	0.0083058
0.0000094	0.039816388	0.0099636

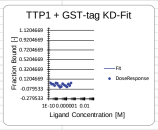

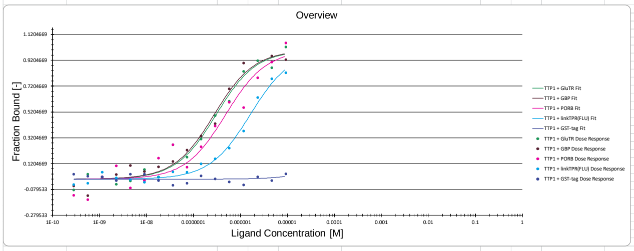

Fig. S6: Statistics of the data shown in Fig. 6. The statistics for data of figure 6 are summarized.

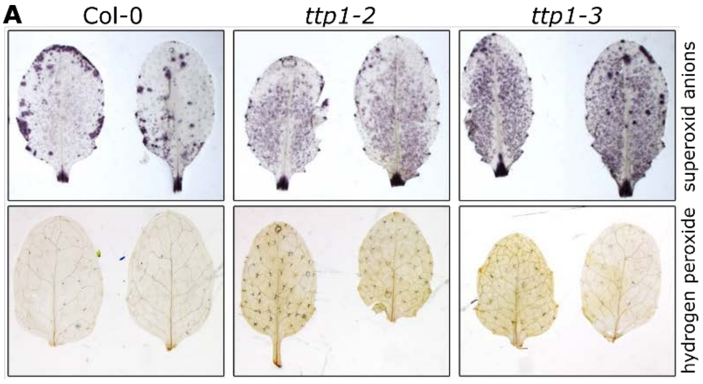

B

		Col-0	*ttp1-2*	*ttp1-3*
common ROS	GST6	a	a	a
$^1O_2$	ATPase	a	a	b
	NOD1	a	a	a
	BAP1	a	a	b
H_2O_2	Ferritin	a	b	b
	cAPX	b	a	b
	tAPX	b	a	a
	sAPX	b	b	a
	PK	a	a	a

0	0.5	1	2	4

Fig. S7: Detection of reactive oxygen species in *ttp1 knockout* and *knockdown* lines. All analyses were performed on four-week-old Arabidopsis plants grown under SD conditions (10 h light / 14 h darkness, 120 µmoles photons m^{-2} s^{-1}, 21°C, 60% humidity). (A) Representative images of leaves stained with nitro-blue tetrazolium chloride or 3,3′-diaminobenzidine to detect superoxide anions or hydrogen peroxide, respectively, are shown. (B) Relative expression levels of the indicated ROS marker genes, which were grouped according to Woodson et al. 2015. The relative expression levels are given below the Table. Statistical significance was calculated by two-way ANOVA analysis (Tukey post-hoc test, $p < 0.05$, n = 4).

name	identifier	TPRpred	% TPR	ChloroP	length TPR domain	number of motifs	similarity	identity	gaps	TM
SG1	At3g18420	4.7E-25	100%	0.570	176 aa	5	49%	26%	3%	no
TTP2	At4g39470	1.8E-19	100%	0.510	175 aa	6	49%	25%	5%	no
TTP1	At1g78915	2.4E-22	100%	0.540	187 aa	5	45%	26%	3%	yes
TTP3	At5g02590	3.3E-23	100%	0.519	210 aa	6	41%	25%	4%	no
TTP4	At3g53560	3.5E-20	100%	0.575	177 aa	5	46%	23%	4%	no
	At2g37400	1.5E-21	100%	0.580	211 aa	7	40%	22%	3%	no
	At3g09490	1.2E-23	100%	0.563	211 aa	6	35%	19%	2%	no
	At4g37210	9.1E-18	100%	Cell-PLoc 2.0	236 aa	3	33%	19%	22%	no
FLU	At3g14110	4.8E-15	99.99%	Cell-PLoc 2.0	72 aa	3	28%	16%	38%	yes
	At1g15290	2.1E-15	99.99%	Cell-PLoc 2.0	202 aa	5	32%	16%	14%	no
	At3g16760	6.3E-22	100%	0.563	108 aa	3	25%	15%	42%	no
	At5g10090	0.0E+00	100%	0.521	336 aa	9	26%	14%	45%	no
	At3g26580	8.9E-12	99.70%	Cell-PLoc 2.0	72 aa	2	18%	12%	62%	yes
	At3g05625	1.0E-11	99.68%	0.545	68 aa	2	20%	11%	63%	no
	At4g10840	0.0E+00	100%	0.582	410 aa	10	20%	11%	55%	no
	At2g31240	0.0E+00	100%	0.540	528 aa	13	16%	10%	65%	no
	At5g53080	1.6E-36	100%	0.567	414 aa	10	18%	10%	56%	no
LPA	At1g02910	1.6E-12	99.87%	0.534	71 aa	2	16%	9%	62%	no

Table S1: List of chloroplast localized TPR-proteins and comparison of the identified TPR-motif to the TPR-motif of Pitt. Overall, 22 TPR proteins with a putative localization in the chloroplast were identified. The localization is based on *in-silico* predictions, made by either ChloroP, Cell-PLoc 2.0 or LOCALIZER (marked with an asterisk, https://localizer.csiro.au/). The TPR motifs were verified by TPRpred (https://toolkit.tuebingen.mpg.de/tools/tprpred). The calculated *P*-value and probability for this prediction is given. The lengths of the identified TPR domain and the numbers of motifs were also calculated by TPRpred. The degrees of identity and similarity of the predicted amino-acid sequences of the TPR domains to the known TPR domain of Pitt were calculated with Needleman-Wunsch Global Alignment (Needleman and Wunsch, 1970). The presence of a transmembrane domain (TM) was predicted by using the TMHMM 2.0 server (https://services.healthtech.dtu.dk/service.php?TMHMM-2.0)

Table S2: List of the used Oligonucleotides

ID	Primername	Sequence
**genotyping**		
At1g78915	*ttp1-1* LP	TTTTCTGCTCAAGAGCTGACC
	ttp1-1 RP	CTAACGTTATCTTACCGGCCC
At1g78915	*ttp1-2* LP	TCAGTGAATAAAACGCAAGGG
	ttp1-2 RP	ATGTTCGAGACACCATGAAGG
At1g78915	*ttp1-3* LP	CAGTGCTCATCCTGAAGACTTCCG
	ttp1-3 RP	ACAAGGGCCTTAGCTTTGTTTGGT
SALK LB	SALK LBb1.3	TCAGGTTGAGTTACTTCTCGG
GABI LB	GABI LB o8409	ATATTGACCATCATACTCATTGC
**qPCR TBS genes**		
At1g13440	5'GAPDH fwd	TTGGTGACAACAGGTCAAGCA
	5'GAPDH rev	AAACTTGTCGCTCAATGCAATC
At2g28390	SAND fwd	AACTCTATGCAGCATTTGATCCACT
	SAND rev	TGATTGCATATCTTTATCGCCATC
At1g78915	TTP1 fwd	CCAATTCAGGTTGAGTTACT
	TTP1 rev	GCCTGGATGAACATTCTCT
At1g58290	HEMA1 fwd	TTGCTGCCAACAAAGAAGAC
	HEMA1 rev	CCGTCTCCAATGAATCCCTC
At3g14110	FLU fwd	AAGCCATACAGTATCACTCCA
	FLU rev	TCCAGAATCTTCACTTTCCCT
At3g56940	CHL27 fwd	GCTTCTTCTGCCTCTCGGTTTATG
	CHL27 rev	GCCGTGGTTCGGTTTGTCTCG
At4g27440	PORB fwd	TGATTACCCTTCAAAGCGTCTCA
	PORB rev	CAATGTATTCGTGTTCCCGGT
**qPCR ROS marker genes (Woodson *et al.*, 2015)**		
At3g28580	ATPase fwd	GAAGATCGGAAAAGCGTGGAA
	ATPase rev	CCGGGTGGTCCAAACAAAAG
At5g64870	NOD1 fwd	GCTGATGCTGCCTTCTATTCAA
	NOD1 rev	TGCGACAAGTCCCTCTGCA
At3g61190	BAP1 fwd	GTGGGATCGTCAATCTTTCG
	BAP1 rev	GGCCACCGTATCCATCAATC
At5g01600	Ferritin fwd	GATCTCAGACTACATCACCCA
	Ferritin rev	GAATCTTCGGGTTCTTCTGTTT
At1g07890	cAPX fwd	TCGAGAAATACGCTGCTGATG
	cAPX rev	ACACAGAGCATACGTCACAG
At1g77490	tAPX fwd	CAAAGTATTCTACGGGAAAGAAGG
	tAPX rev	TGTTGAGGAAGTAATTTGTGGG
At4g08390	sAPX fwd	GGGCACATAATTTACTCAACCA
	sAPX rev	ATCCTTTGAACAGCCAGAAAC
At3g49160	PK fwd	TCCTGCTTTGGTATGGCTCTG
	PK rev	ACATGAGCAGGAGGTTCTGTTC
At1g78915	TTP1_BamHI fwd	GGCGCCCAGCAAAGGAAATC
	TTP1_SalI rev	CTGCAGTCATAGTTTGGAGTATCTATCC

References Supplement:

Edgar RC. 2004. MUSCLE: multiple sequence alignment with high accuracy and high throughput Nucleic Acids Research **32**, 5.

Jones DT, Taylor WR, Thornton JM. 1992. The rapid generation of mutation data matrices from protein sequences. Comput Appl Biosci **8**, 275-282.

Kumar S, Stecher G, Li M, Knyaz C, Tamura K. 2018. MEGA X: Molecular Evolutionary Genetics Analysis across Computing Platforms. Mol Biol Evol **35**, 1547-1549.

Needleman SB, Wunsch CD. 1970. A general method applicable to the search for similarities in the amino acid sequence of two proteins. Journal of Molecular Biology. **48**, 10.

Woodson JD, Joens MS, Sinson AB, Gilkerson J, Salomé PA, Weigel D, Fitzpatrick JA, Chory J. 2015. Ubiquitin facilitates a quality-control pathway that removes damaged chloroplasts. Science **350**, 450-454.
